# Leveraging multimodal learning for enhanced drug-target interaction prediction

**DOI:** 10.3389/fphar.2025.1639979

**Published:** 2025-11-19

**Authors:** Guo Chen, Kaixin Sun

**Affiliations:** 1 Department of Spine Surgery, Department of Orthopedics, The Seventh Affiliated Hospital, Southern Medical University, Foshan, China; 2 North China Electric Power University, Beijing, China

**Keywords:** multimodal learning, drug-target interaction, molecular encoding, curriculum learning, biomedical data fusion

## Abstract

**Introduction:**

The evolving landscape of artificial intelligence in drug discovery necessitates increasingly sophisticated approaches to predict drug-target interactions (DTIs) with high precision and generalizability. In alignment with the current surge of interest in AI-driven pharmacological modeling and integrative biomedical data analysis, this study introduces a multimodal framework for enhancing DTI prediction by fusing heterogeneous data sources. While conventional methods typically rely on unimodal inputs such as chemical structures or protein sequences, they fall short in capturing the complex, multi-faceted nature of biochemical interactions and are often limited in adaptability across different tasks or incomplete datasets. These limitations impede the model’s capability to generalize beyond narrow benchmarks and reduce interpretability when modalities are missing or noisy.

**Methods:**

To address these challenges, we propose a comprehensive multimodal learning pipeline composed of three principal innovations. The Unified Multimodal Molecule Encoder (UMME) jointly embeds molecular graphs, textual descriptions, transcriptomics, protein sequences, and bioassay data using modality-specific enc followed by a hierarchical attention-based fusion strategy. This encoder is capable of aligning intra- and inter-modal representations while retaining high-level semantic features critical for interaction prediction. We introduce a robust training strategy named Adaptive Curriculum-guided Modality Optimization (ACMO), which dynamically prioritizes more reliable or informative modalities during early training and gradually incorporates less certain data via a curriculum mechanism. This allows the model to maintain strong performance even when faced with modality absence or noise, thereby mimicking realistic drug screening conditions. We employ a novel cross-modal contrastive alignment loss and modality dropout scheduling, which together enforce consistency and encourage generalization across diverse data settings.

**Results:**

Experiments on multiple benchmark datasets demonstrate that our framework achieves state-of-the-art performance in drug-target affinity estimation and binding prediction tasks, particularly under conditions of partial data availability.

**Discussion:**

Ablation studies confirm the effectiveness of both UMME and ACMO components in improving accuracy and robustness.

## 1 Introduction

In recent years, the accurate prediction of drug-target interactions (DTIs) has emerged as a critical research objective in computational biology and drug discovery. With the explosion of biological data and chemical information, there is an increasing need for methods that can efficiently integrate heterogeneous sources of information (Lin et al., 2024). Traditional single-modal approaches are often limited in their capacity to capture the complex nature of biological systems (Han et al., 2024). In contrast, multimodal learning provides a framework that not only integrates diverse data types such as molecular structures, protein sequences, and biological networks, but also enhances the robustness and generalizability of DTI models (Wei and Hu, 2024). By combining complementary information from various modalities, multimodal learning not only enables better understanding of biological mechanisms, but also facilitates drug repurposing and accelerates the discovery of novel therapeutics (Peng et al., 2022).

Earlier efforts to predict DTIs relied on interpretable inference mechanisms grounded in chemical heuristics and sequence-based descriptors (Hu et al., 2023). These models were typically constructed by defining similarity measures or interaction scores based on manually engineered molecular or protein features (Zong et al., 2023). While they offered biological insight and were relatively easy to interpret, their expressiveness was often constrained by simplistic assumptions and fixed rules (Xu et al., 2022). As a result, they struggled to generalize in scenarios involving noisy data or novel compounds with unknown structural variations (Xu et al., 2023). Subsequent progress in DTI prediction came from models capable of learning patterns from data more adaptively through statistical optimization and algorithmic flexibility. Approaches such as kernel-based classifiers, ensemble learners, and factorization-based systems began to replace static rule-based pipelines (Wang et al., 2023). These techniques improved performance by leveraging more informative combinations of features, offering better scalability and less dependence on handcrafted descriptors (Wei et al., 2023). Nevertheless, they still exhibited limited depth in capturing the sequential and relational complexity embedded in molecular and biological inputs, often requiring extensive preprocessing steps and manual curation of input features (Hao et al., 2022). Their representational power, while stronger than early methods, was not sufficient for fully modeling the intricate biochemical interactions in DTI tasks (Song et al., 2023).

The most recent advances have incorporated neural architectures capable of directly consuming high-dimensional raw inputs and extracting multilevel abstractions (Zhang et al., 2023). Neural models such as graph-based encoders, convolutional extractors, and sequence-attention mechanisms are now frequently deployed to capture structural and contextual dependencies within and across molecules and proteins (Martínez-Maldonado et al., 2023). These architectures enable end-to-end learning pipelines and support transferability via pretraining on large biological corpora. However, many of these models still operate within single-input modalities and often sacrifice transparency for performance (Joseph et al., 2023). To move beyond these limitations, recent trends have begun integrating multimodal strategies with neural backbones, allowing for both hierarchical feature learning and biological relevance through cross-modal interactions (Zhou et al., 2023). This evolution sets the stage for more comprehensive and interpretable DTI prediction systems (Zhao et al., 2023).

To address the aforementioned limitations of single-modal and deep learning-based DTI models, we propose a novel multimodal learning framework that integrates diverse biological and chemical modalities in a unified architecture. This approach is designed to leverage complementary features from multiple sources, including molecular graphs, protein sequences, 3D structures, and biological interaction networks. By jointly modeling these data types, our framework aims to capture both local interaction patterns and global biological context. Furthermore, cross-modal attention mechanisms and fusion strategies enable the model to dynamically prioritize the most informative features, improving both prediction accuracy and interpretability. This integrative strategy not only enhances robustness against data sparsity but also opens new avenues for identifying off-target effects and repositioning existing drugs. Through rigorous benchmarking on multiple DTI datasets, we demonstrate that our multimodal method consistently outperforms state-of-the-art baselines and generalizes well to novel drug-target pairs, validating the effectiveness of the proposed framework.

The proposed method has several key advantages:

• We introduce a novel multimodal fusion module that enables the integration of heterogeneous biological and chemical data in a unified representation space.
• The method features adaptive attention mechanisms that dynamically weight different modalities, allowing for efficient, context-aware prediction across diverse biological scenarios.
• Extensive experiments on benchmark DTI datasets show superior performance over existing methods, achieving improvements in both precision and recall, particularly in low-resource settings.

## 2 Related work

### 2.1 From single-modal encoding to multimodal integration

Traditional drug-target interaction (DTI) prediction models largely rely on unimodal inputs such as molecular fingerprints or protein descriptors (Shi et al., 2022; Bayoudh et al., 2021). These approaches, though interpretable, offer limited expressiveness due to their inability to model the intricate interactions across biological and chemical domains. With the emergence of deep learning, data-driven architectures like graph neural networks (GNNs) for molecular graphs (Lian et al., 2022) and convolutional neural networks (CNNs) or recurrent neural networks (RNNs) for protein sequences (Ma et al., 2021) have improved the performance of DTI models. Methods such as DeepDTA and WideDTA introduced sequence-level fusion via concatenated embeddings (Zhang et al., 2022), but still treated drugs and targets independently during early encoding stages. In recent years, multimodal learning frameworks have been developed to address these limitations by integrating multiple sources of biomedical data—ranging from SMILES strings and 3D conformations to gene expression profiles and assay results. For instance, the MDTips framework developed by Xia et al. titled *MDTips: a multimodal-data-based drug–target interaction prediction system fusing knowledge, gene expression profile, and structural data* (Xia et al., 2023), and the MFFDTA model proposed by Wang et al., in 2024, titled *MFFDTA: A Multimodal Feature Fusion Framework for Drug-Target Affinity Prediction* (Wang et al., 2024), integrate diverse data modalities into unified prediction pipelines, thereby improving representational fidelity and predictive robustness. These models demonstrate that fusing structural, expression-based, and knowledge-driven features leads to significantly improved generalization and performance in drug-target affinity tasks.

### 2.2 Hierarchical and attention-based fusion strategies

Attention mechanisms play a pivotal role in recent multimodal DTI prediction architectures by enabling dynamic weighting of heterogeneous inputs. Unlike static fusion schemes, co-attention and self-attention mechanisms can capture the contextual relevance of each modality during inference (Du et al., 2022). Examples such as AttentionDTA and MONN (Fan et al., 2022; Chango et al., 2022) show that modeling mutual attention between drug and protein sequences effectively uncovers interaction-specific substructures. More recent designs utilize hierarchical attention to model both intra- and inter-modal dependencies, leading to better interpretability and scalability (Tian et al., 2024). Transformer-based encoders have also been successfully adapted from natural language processing to biomedical settings. GraphTransformer and MolTrans, for example, leverage multi-head self-attention to jointly reason over molecular and protein contexts (Ektefaie et al., 2022). However, the increased model complexity brings potential overfitting and computational cost, particularly in high-dimensional or sparse data settings (Yan et al., 2022). To mitigate these risks, newer models adopt curriculum-guided fusion, as in CCL-ASPS (Tian et al., 2024), which gradually introduces modalities during training based on their reliability.

### 2.3 Contrastive learning and pretrained representation alignment

Pretrained models offer a powerful solution for capturing biochemical semantics from large-scale unlabeled datasets. ChemBERTa and GROVER (Zong et al., 2024) are commonly used for encoding molecular graphs or SMILES, while ProtBERT and ESM (Zhang D.-E. et al., 2025) provide protein sequence representations enriched with evolutionary signals. These embeddings, when integrated into multimodal pipelines, significantly enhance the performance of DTI prediction. Nevertheless, aligning latent spaces across modalities remains challenging due to representational mismatches. To address this, models like DrugBAN and BioTrans employ cross-modal contrastive learning objectives (Xing et al., 2021), while others apply projection layers or distance-based regularization techniques (Xing et al., 2017). The work by Zhu Sidan and Luo Dixin in 2024, titled *Enhancing Multi-modal Contrastive Learning* via *Optimal Transport-Based Consistent Modality Alignment* (Zhu and Luo, 2024), further extends this idea by incorporating optimal transport-based regularization to ensure consistent cross-modal alignment. Moreover, strategies such as task-conditioned attention modulation and uncertainty-aware scheduling have proven effective in dynamically weighting each modality’s contribution (Wang et al., 2024). Such mechanisms ensure that noisy or sparse data sources do not overwhelm the learning process, which is particularly valuable in real-world clinical scenarios where modality availability can vary.

Despite the notable progress in multimodal DTI prediction, several key challenges remain unresolved. First, most existing models assume the availability of complete modalities during training and inference, which is often not the case in real-world biomedical settings where some inputs may be missing or noisy. Second, there is limited incorporation of uncertainty modeling and modality-specific reliability, making many architectures sensitive to modality imbalance or degradation. Third, while hierarchical or transformer-based fusion strategies have been explored, they are often static and lack adaptability to task-specific or sample-specific conditions. These limitations motivate the core design objectives of the present study. The proposed UMME (Unified Multimodal Molecule Encoder) introduces hierarchical attention to support flexible intra- and inter-modality representation learning, while ACMO (Adaptive Curriculum-guided Modality Optimization) dynamically regulates modality contribution based on uncertainty and training progression. Together, these components address the gap between rigid fusion pipelines and the need for robust, adaptive, and interpretable multimodal models, particularly in the presence of incomplete or unreliable data. This integration positions the proposed framework to offer both practical scalability and enhanced biological fidelity in DTI prediction tasks.

## 3 Methods

### 3.1 Overview

Multimodal drug discovery represents a new paradigm in pharmaceutical research that leverages heterogeneous data sources to enable more accurate, robust, and generalizable predictive modeling for tasks such as molecular property prediction, drug-target interaction analysis, and lead compound optimization. Traditional drug discovery pipelines often rely on unimodal data—typically molecular graphs, chemical structures, or omics data—limiting their capacity to capture the complex, high-dimensional relationships underlying drug mechanisms. In contrast, the multimodal approach synergistically integrates diverse modalities, such as molecular graphs, textual chemical descriptions, biological assay profiles, structural data, and even biomedical images, to enable richer representation learning and improved downstream performance. This section introduces the methodology proposed in this paper, which builds upon the recent success of multimodal representation learning in computational biology. We divide our method section into three integral components that work in concert to construct a robust, interpretable, and scalable framework for drug discovery.

In Section 3.2, we the formal problem setting and provide the mathematical foundation necessary to understand the multimodal integration problem. We define the types of modalities considered, the notational conventions, and the inter-modality alignment mechanisms, which set the stage for the subsequent architectural innovations. This section also outlines the rationale for multimodal learning in the context of biological data, highlighting the types of statistical dependencies that can emerge when combining modalities such as gene expression and molecular structure. In Section 3.3, we detail the design and implementation of our novel model architecture, named Unified Multimodal Molecule Encoder (UMME). UMME is built upon a joint encoder-decoder paradigm, in which modality-specific encoders are used to project each input type into a shared latent space, followed by a modality fusion module that aggregates cross-modal signals into a cohesive representation. This representation is subsequently used for downstream tasks such as drug-target affinity prediction, toxicity classification, and bioactivity estimation. The architecture is characterized by hierarchical attention, cross-modal contrastive loss, and alignment-aware embeddings, allowing it to capture both intra-modal semantic features and inter-modal relational structure. In Section 3.4, a novel strategy is described for robust training and generalization under the constraints of missing modalities and noisy data, termed Adaptive Curriculum-guided Modality Optimization (ACMO). This strategy is designed to address a central challenge in multimodal learning: the incomplete observation of modalities across datasets. ACMO dynamically adjusts the importance of each modality during training, gradually increasing fusion complexity as learning progresses. The curriculum-inspired design ensures that simpler unimodal representations are mastered before higher-order multimodal interactions are introduced, thereby improving convergence stability and resilience to missing data. Through the combination of these components, our proposed framework achieves state-of-the-art performance on multiple benchmarks for multimodal drug discovery. Furthermore, ablation studies demonstrate the complementary contributions of modality alignment, hierarchical fusion, and adaptive curriculum strategies. Collectively, this section offers a comprehensive introduction to the algorithmic core of our approach and lays the groundwork for detailed discussion in the following sections.

### 3.2 Preliminaries

In this section, we provide a formal foundation for the problem of multimodal drug discovery, by constructing a symbolic framework that encapsulates the central modeling objectives, data characteristics, and inter-modality dependencies. We aim to formulate the multimodal setting in a way that captures the inherent heterogeneity, alignment challenges, and predictive tasks relevant to drug development pipelines.

Let $D={\left\{\left(x_{1}^{\left(i\right)},x_{2}^{\left(i\right)},\ldots ,x_{K}^{\left(i\right)},y^{\left(i\right)}\right)\right\}}_{i=1}^{N}$ denote the multimodal dataset, where $x_{k}^{\left(i\right)}\in M_{k}$ is the i -th sample from the k -th modality, and $y^{\left(i\right)}$ is the supervised label, such as drug efficacy, toxicity, or binding affinity.

Our goal is to learn a function $F:M\to R^{q}$ that maps a multimodal input tuple to a prediction space, such that Equation 1:

$${\hat{y}}^{\left(i\right)}=F\left(x_{1}^{\left(i\right)},x_{2}^{\left(i\right)},\ldots ,x_{K}^{\left(i\right)}\right)$$ (1)

To enable this, we define a modality-specific encoder $\phi_{k}:M_{k}\to R^{d_{k}}$ that transforms raw input $x_{k}$ into a latent representation $z_{k}=\phi_{k}\left(x_{k}\right)$ . The full latent representation is the concatenation or aggregation (Equation 2):

$$Z=\Psi \left(z_{1},z_{2},\ldots ,z_{K}\right),\;\text{where }z_{k}=\phi_{k}\left(x_{k}\right)$$ (2)

Ψ may be a simple concatenation $\Psi \left(z_{1},\ldots ,z_{K}\right)=\left[z_{1}\|z_{2}\|\cdots \|z_{K}\right]$ or a learned cross-modal fusion operator.

Given the multimodal input structure, the challenges lie in the heterogeneity, incomplete modality observation, and semantic alignment across modalities. We now formalize these notions.

Molecular graphs are defined as G=(V,E) , where each node v∈V has a feature vector $x_{v}\in R^{d_{v}}$ and each edge (u,v)∈E has a bond-type feature $e_{uv}\in R^{d_{e}}$ . We denote the graph embedding by Equation 3:

$$z_{1}=\phi_{1}\left(G\right)={\text{AGGREGATE}}_{v\in V}\left(f_{\text{node}}\left(x_{v}\right)+\underset{u\in N\left(v\right)}{\sum }f_{\text{edge}}\left(e_{uv}\right)\right)$$ (3)

Here, $f_{\text{node}}$ and $f_{\text{edge}}$ are learnable functions and N(v) denotes the neighborhood of node v .

For SMILES strings $S=\left[s_{1},s_{2},\ldots ,s_{n}\right]$ , we define a tokenizer $T:\Sigma^{*}\to R^{n\times d_{s}}$ and encode it via a transformer $T_{\theta }$ (Equation 4):

$$z_{2}=\phi_{2}\left(S\right)=\text{Pooling}\left(T_{\theta }\left(T\left(S\right)\right)\right)$$ (4)

Similarly, protein sequences $P=\left[p_{1},p_{2},\ldots ,p_{m}\right]\in A^{m}$ are mapped via a pretrained language model over protein space (Equation 5):

$$z_{4}=\phi_{4}\left(P\right)=\text{Pooling}\left({BERT}_{\text{prot}}\left(P\right)\right)$$ (5)

High-dimensional numeric modalities, such as transcriptomic data $T\in R^{d_{T}}$ and assay data $B\in R^{d_{B}}$ , are directly embedded via fully connected transformations (Equations 6, 7):

$$z_{3}=\phi_{3}\left(T\right)=\sigma \left(W_{T}T+b_{T}\right)$$ (6)

$$z_{5}=\phi_{5}\left(B\right)=\sigma \left(W_{B}B+b_{B}\right)$$ (7)

To enforce alignment between representations across modalities, we define an auxiliary pairwise alignment operator $A:R^{d_{i}}\times R^{d_{j}}\to R$ (Equation 8):

$$A\left(z_{i},z_{j}\right)=\frac{z_{i}^{⊤}z_{j}}{\left\|z_{i}\|\cdot \|z_{j}\right\|}$$ (8)

Given a reference modality $z_{1}$ and a support modality $z_{k}$ , we require (Equation 9):

$$E_{\left(x_{1},x_{k}\right)}\left[A\left(z_{1},z_{k}\right)\right]\geq \tau$$ (9)

where τ is a tunable alignment margin.

Once individual modalities are embedded, we define a fusion function $\Psi :R^{d_{1}}\times \cdots \times R^{d_{K}}\to R^{d}$ and a predictor $F_{\theta }:R^{d}\to R^{q}$ such that Equation 10:

$$Z=\Psi \left(z_{1},z_{2},\ldots ,z_{K}\right),\;\hat{y}=F_{\theta }\left(Z\right)$$ (10)

In practice, Ψ may use attention or gating mechanisms (Equation 11):

$$Z=\overset{K}{\underset{k=1}{\sum }}\alpha_{k}z_{k},\;\alpha_{k}=\frac{\exp\left(w^{⊤}z_{k}\right)}{\sum_{j}\exp\left(w^{⊤}z_{j}\right)}$$ (11)

Textual descriptions used in this framework include various types of structured and unstructured annotations that accompany chemical and biological entities. For chemical compounds, these descriptions comprise IUPAC names, mechanism of action summaries, pharmacological class labels, and clinical usage indications as curated in DrugBank and ChEMBL databases. For proteins, the textual input may include functional summaries, subcellular localization notes, and disease associations derived from UniProt and Gene Ontology annotations. Free-text metadata such as compound warnings, side-effect narratives, and therapeutic summaries extracted from biomedical literature are included when available. These textual fields are tokenized using a domain-specific tokenizer and subsequently encoded via a pretrained transformer-based language model (BioBERT or SciBERT). This modality is designed to provide contextual and semantic background information that complements the structural features of molecular graphs and the sequential features of proteins or SMILES strings. Textual inputs play a particularly important role in scenarios where explicit structural or assay data may be sparse or noisy. By incorporating this modality, the model gains access to curated expert knowledge and published biomedical insights that may not be encoded in purely numerical or symbolic forms.

### 3.3 Unified Multimodal Molecule Encoder (UMME)

**FIGURE 1:**
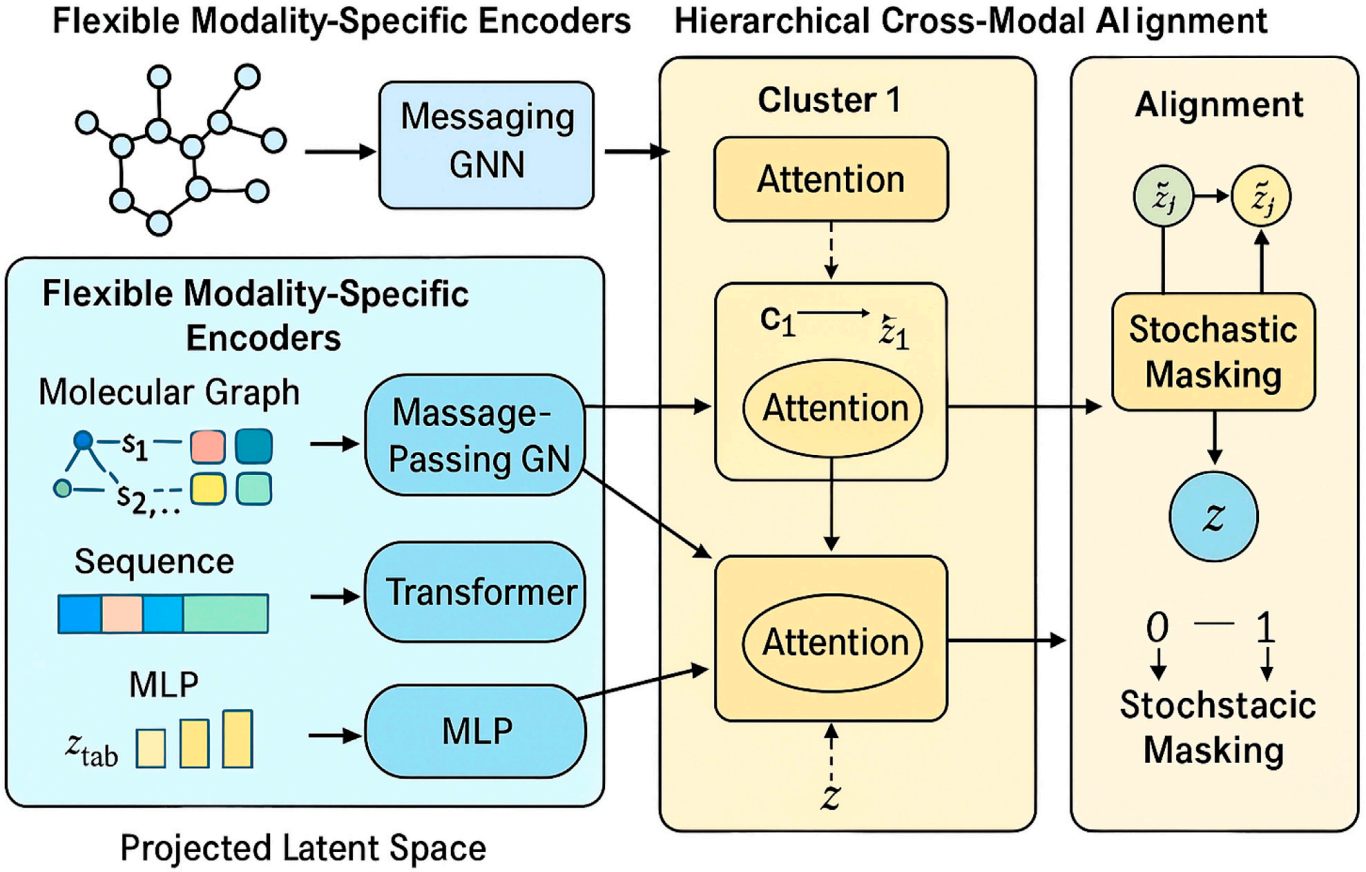
Schematic diagram of the Unified Multimodal Molecule Encoder (UMME) framework. The model consists of three main modules: Flexible Modality-Specific Encoders for handling different biomedical inputs (molecular graphs, SMILES sequences, and omics profiles); Hierarchical Cross-Modal Fusion, which aggregates semantically grouped modalities via local anxd global attention; and Modality Robustness and Alignment module, which introduces stochastic masking and contrastive alignment loss to ensure robustness under partial modality settings.

We propose the Unified Multimodal Molecule Encoder (UMME), a novel neural framework for encoding heterogeneous biomedical data into a unified latent representation. UMME is designed to flexibly handle diverse data types—including molecular graphs, sequences, and numeric profiles—while supporting missing modality scenarios. Below, we highlight three primary innovations of the UMME architecture. Figure 1 illustrates the overall architecture of the UMME framework, where molecular graphs, protein sequences, and omics profiles are independently encoded and hierarchically fused into a unified latent representation.

#### 3.3.1 Flexible Modality-specific encoders

**FIGURE 2:**
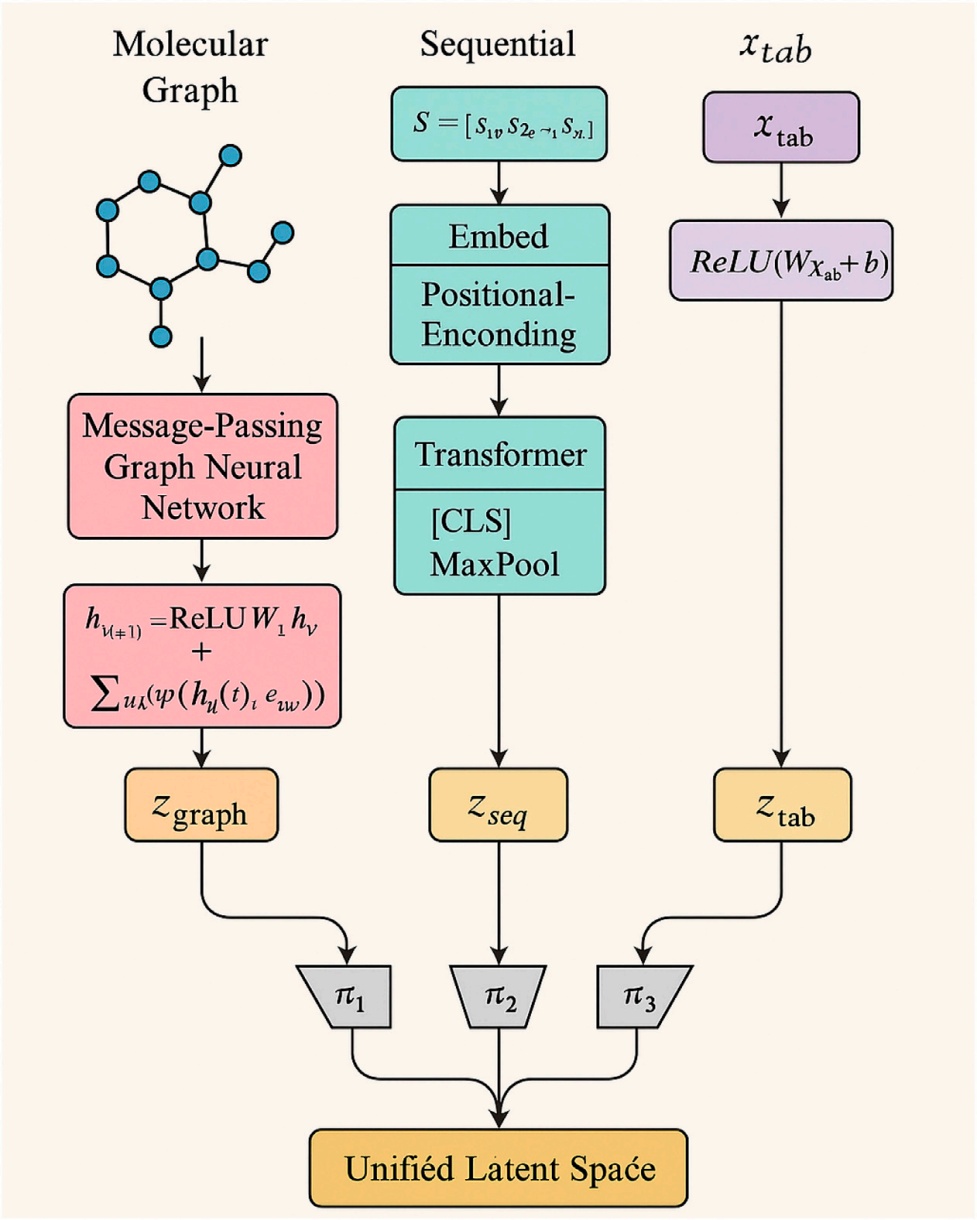
Schematic diagram of Flexible Modality-Specific Encoders. The diagram illustrates the encoding pathways for different input modalities used in this study. Molecular graphs are processed by message-passing graph neural networks, sequential data (such as SMILES or protein sequences) are handled by transformer-based encoders, and tabular features (transcriptomic or assay data) are encoded via fully connected networks. All modality-specific embeddings are projected into a unified latent space for multimodal fusion.

UMME integrates a suite of specialized encoders tailored for different types of biomedical inputs, enabling rich and modality-aware feature extraction. Each input modality—be it structural, sequential, or numerical—is first processed by a dedicated neural encoder designed to best capture the unique characteristics of the data. For molecular graphs, which represent atoms as nodes and chemical bonds as edges, UMME employs a message-passing graph neural network. Figure 2 further details the modality-specific encoders, showing how graph neural networks, transformer encoders, and fully connected layers are applied to different input types.

This GNN propagates information across atom neighborhoods and updates each node embedding through a learnable message aggregation function (Equation 12):

$$h_{v}^{\left(t+1\right)}=\text{ReLU}\left(W_{1}h_{v}^{\left(t\right)}+\underset{u\in N\left(v\right)}{\sum }\psi \left(h_{u}^{\left(t\right)},e_{uv}\right)\right),$$ (12)

where $h_{v}^{\left(t\right)}$ is the hidden representation of node v at layer t and ψ is a learnable edge-aware message function. The final graph embedding is then pooled across all nodes (Equation 13):

$$z_{\text{graph}}=\text{READOUT}\left({\left\{h_{v}^{\left(T\right)}\right\}}_{v\in V}\right),$$ (13)

where READOUT could be sum, mean, or attention-based pooling. For sequential modalities such as SMILES strings or protein amino acid chains, a transformer encoder is employed to capture long-range dependencies and contextual embeddings. Given an input sequence $S=\left[s_{1},s_{2},\ldots ,s_{n}\right]$ , we first apply token embedding and positional encoding (Equation 14):

$$E=\text{Embed}\left(S\right)+\text{PositionalEncoding}\left(S\right),$$ (14)

and then pass it through a multi-layer transformer to obtain hidden states H=Transformer(E) . A sequence-level representation is extracted using a combination of the [CLS] token and max pooling (Equation 15):

$$z_{\text{seq}}=\text{CLS}\left(H\right)+\text{MaxPool}\left(H\right).$$ (15)

For tabular or vectorial inputs, such as gene expression profiles or molecular fingerprints, a fully connected neural network with ReLU activations and dropout regularization is used. Let $x_{\text{tab}}$ be the input vector, then (Equation 16):

$$z_{\text{tab}}=\text{ReLU}\left(Wx_{\text{tab}}+b\right),$$ (16)

where W and b are learned parameters. After modality-specific encoding, all embeddings $z_{k}$ are projected into a unified latent space via learned linear mappings $\pi_{k}$ (Equation 17):

$${\tilde{z}}_{k}=\pi_{k}\left(z_{k}\right)\in R^{d},$$ (17)

allowing downstream modules to process multimodal inputs within a consistent representation space. This architecture not only accommodates heterogeneous data but also ensures that each modality is optimally encoded according to its structural nature, improving both the expressiveness and flexibility of the overall system.

#### 3.3.2 Hierarchical cross-modal fusion

To capture complex interactions among heterogeneous data sources while preserving semantic coherence, UMME employs a hierarchical cross-modal fusion mechanism. This design enables the model to integrate multimodal embeddings in a way that respects the intrinsic relationships among modalities, avoids overfitting to dominant sources, and facilitates both local and global representation learning. The fusion process unfolds in two stages: an intra-cluster attention aggregation among semantically similar modalities and a global-level attention across clusters to produce the final unified embedding.

At the first level, all input modalities are grouped into predefined semantic clusters ${\left\{C_{j}\right\}}_{j=1}^{m}$ , based on structural similarity or data type affinity—for example, grouping molecular structure and chemical fingerprint into one cluster, and protein sequences and gene expression vectors into another. For each cluster $C_{j}$ , a learned attention mechanism aggregates the projected modality embeddings ${\tilde{z}}_{k}$ within the group. The attention weights are computed using a query vector $q_{j}$ shared across modalities in that cluster (Equation 18):

$$\alpha_{k}^{\left(j\right)}=\frac{\exp\left(q_{j}^{⊤}{\tilde{z}}_{k}\right)}{\sum_{k^{\prime }\in C_{j}}\exp\left(q_{j}^{⊤}{\tilde{z}}_{k^{\prime }}\right)},\;u_{j}=\underset{k\in C_{j}}{\sum }\alpha_{k}^{\left(j\right)}{\tilde{z}}_{k},$$ (18)

where $\alpha_{k}^{\left(j\right)}$ denotes the importance of modality k in cluster j , and $u_{j}$ represents the cluster-level embedding. This intra-group attention ensures that modalities within the same semantic group can adaptively contribute based on contextual relevance, such as favoring structural embeddings for chemical similarity tasks or sequence-based encoders for protein binding predictions.

Once cluster-level vectors ${\left\{u_{j}\right\}}_{j=1}^{m}$ are obtained, they are concatenated and fed into a transformer encoder that models the inter-cluster dependencies. The transformer captures hierarchical relationships and contextual influences across distinct modality groups, leveraging self-attention layers to dynamically reweight the contribution of each cluster. Let $U=\left[u_{1},u_{2},\ldots ,u_{m}\right]$ be the matrix of fused cluster embeddings, then the global representation is computed as Equation 19:

$$H={\text{Transformer}}_{\text{global}}\left(U\right),\;z=\text{Concat}\left(\text{CLS}\left(H\right),\text{Mean}\left(H\right)\right),$$ (19)

where CLS(H) serves as the aggregated global summary vector, and Mean(H) ensures holistic averaging across clusters. This dual pooling approach provides both task-oriented and distribution-aware perspectives on the final representation.

Notably, this hierarchical design offers several advantages. It provides modularity, allowing the model to flexibly incorporate new modalities or remove noisy sources without retraining the entire system. By decomposing fusion into local and global steps, it prevents early over-mixing of incompatible features, improving interpretability and stability. It serves as a natural interface for incorporating attention masks, curriculum pacing, or task-specific routing schemes—thus supporting extensions such as Adaptive Curriculum-guided Modality Optimization (ACMO). The hierarchical cross-modal fusion in UMME enables the model to reason over multimodal inputs with both structural precision and semantic awareness.

#### 3.3.3 Modality robustness and alignment

UMME is explicitly designed to function under the practical constraints of incomplete, noisy, or partially missing biomedical modalities, which are prevalent in real-world clinical and omics datasets. To this end, the framework incorporates a dual strategy—modality-aware attention masking and contrastive alignment regularization—to promote robustness and semantic coherence in multimodal learning. UMME introduces stochastic modality dropout during training, in which certain input modalities are randomly masked to simulate missing data. For each modality k , a binary indicator variable $\delta_{k}\in \left\{0,1\right\}$ denotes its availability in a given instance. This masking is applied directly to the attention mechanism in the intra-cluster fusion process, reweighting the contribution of available modalities based on their learned importance. The masked attention is computed as Equation 20:

$$\alpha_{k}^{\left(j\right)}=\frac{\delta_{k}\cdot \exp\left(q_{j}^{⊤}{\tilde{z}}_{k}\right)}{\sum_{k^{\prime }}\delta_{k^{\prime }}\cdot \exp\left(q_{j}^{⊤}{\tilde{z}}_{k^{\prime }}\right)},$$ (20)

where $q_{j}$ is the cluster-level query vector and ${\tilde{z}}_{k}$ is the projected embedding of modality k . This formulation ensures that only present modalities participate in fusion, and that their relative contributions are still modulated by learned attention weights. During training, $\delta_{k}$ is sampled from a Bernoulli distribution to enforce stochastic regularization, while during inference, it reflects actual data availability.

In parallel, UMME enforces semantic consistency across modalities by introducing a contrastive alignment loss. The goal is to ensure that embeddings derived from different modalities of the same sample encode congruent biological information in the shared latent space. Given two modality embeddings $z_{i}$ and $z_{j}$ for the same instance, the model is encouraged to bring them closer in representation space, while simultaneously pushing them away from unrelated samples (negatives) within the batch. This is implemented using a symmetric InfoNCE-style loss (Equation 21):

$$L_{\text{align}}=-\log\frac{\exp\left(\text{sim}\left(z_{i},z_{j}\right)/\tau \right)}{\sum_{k}\exp\left(\text{sim}\left(z_{i},z_{k}^{-}\right)/\tau \right)},$$ (21)

where $\text{sim}\left(a,b\right)=\frac{a^{⊤}b}{\left\|a\|\|b\right\|}$ denotes cosine similarity, τ is a temperature parameter, and $z_{k}^{-}$ are embeddings of negative pairs drawn from other samples in the same batch. This formulation encourages intra-sample agreement across modalities while preserving inter-sample separability. It is particularly effective for aligning modalities with different representational geometries, such as molecular graphs and gene expression profiles.

Together, these techniques allow UMME to gracefully degrade under partial observation and maintain high-quality representations despite missing or unreliable inputs. The contrastive loss acts as a semantic bridge across modality boundaries, while the masked attention mechanism ensures resilience in the presence of data sparsity. Importantly, this design allows UMME to be trained once and deployed in settings with variable modality availability, including zero-shot generalization to unseen modality subsets. By explicitly modeling uncertainty and enforcing cross-modal consistency, the architecture achieves strong generalization, enhanced interpretability, and robust performance across diverse biomedical tasks.

### 3.4 Adaptive curriculum-guided modality optimization (ACMO)

**FIGURE 3:**
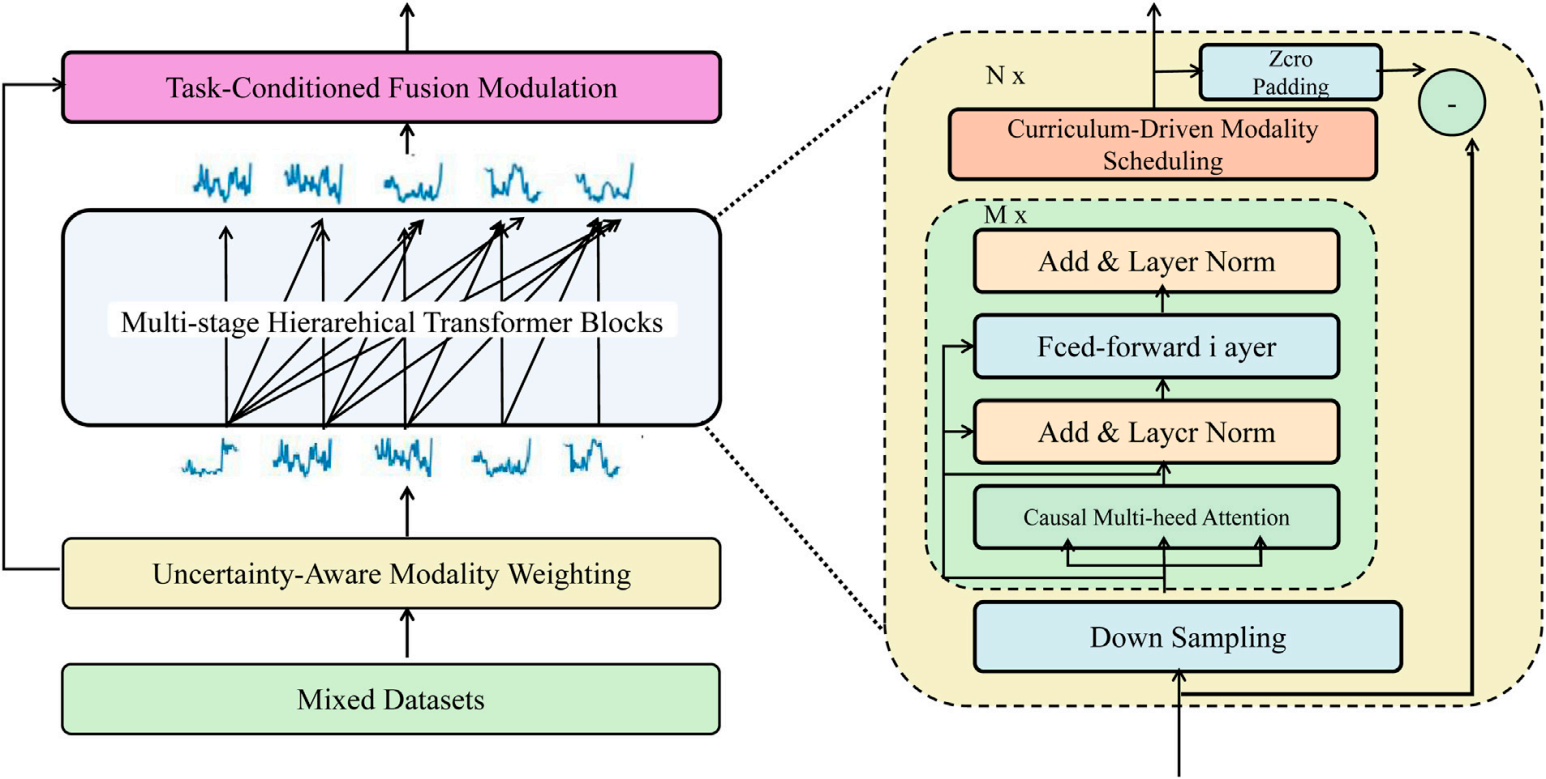
Schematic diagram of the ACMO framework. The architecture illustrates the core components of Adaptive Curriculum-guided Modality Optimization, including uncertainty-aware modality weighting, curriculum-driven modality scheduling, and task-conditioned fusion modulation, orchestrated through hierarchical transformer blocks to adaptively regulate information flow across heterogeneous biomedical modalities.

To enhance multimodal learning under realistic biomedical constraints—such as noisy signals, missing data, and variable modality informativeness—we propose Adaptive Curriculum-guided Modality Optimization (ACMO). This framework dynamically adjusts training emphasis across modalities based on confidence, task utility, and training progression. Figure 3 outlines the ACMO training strategy, which adaptively manages the inclusion of modalities through uncertainty-aware weighting and curriculum-guided scheduling.

#### 3.4.1 Uncertainty-aware modality weighting

**FIGURE 4:**
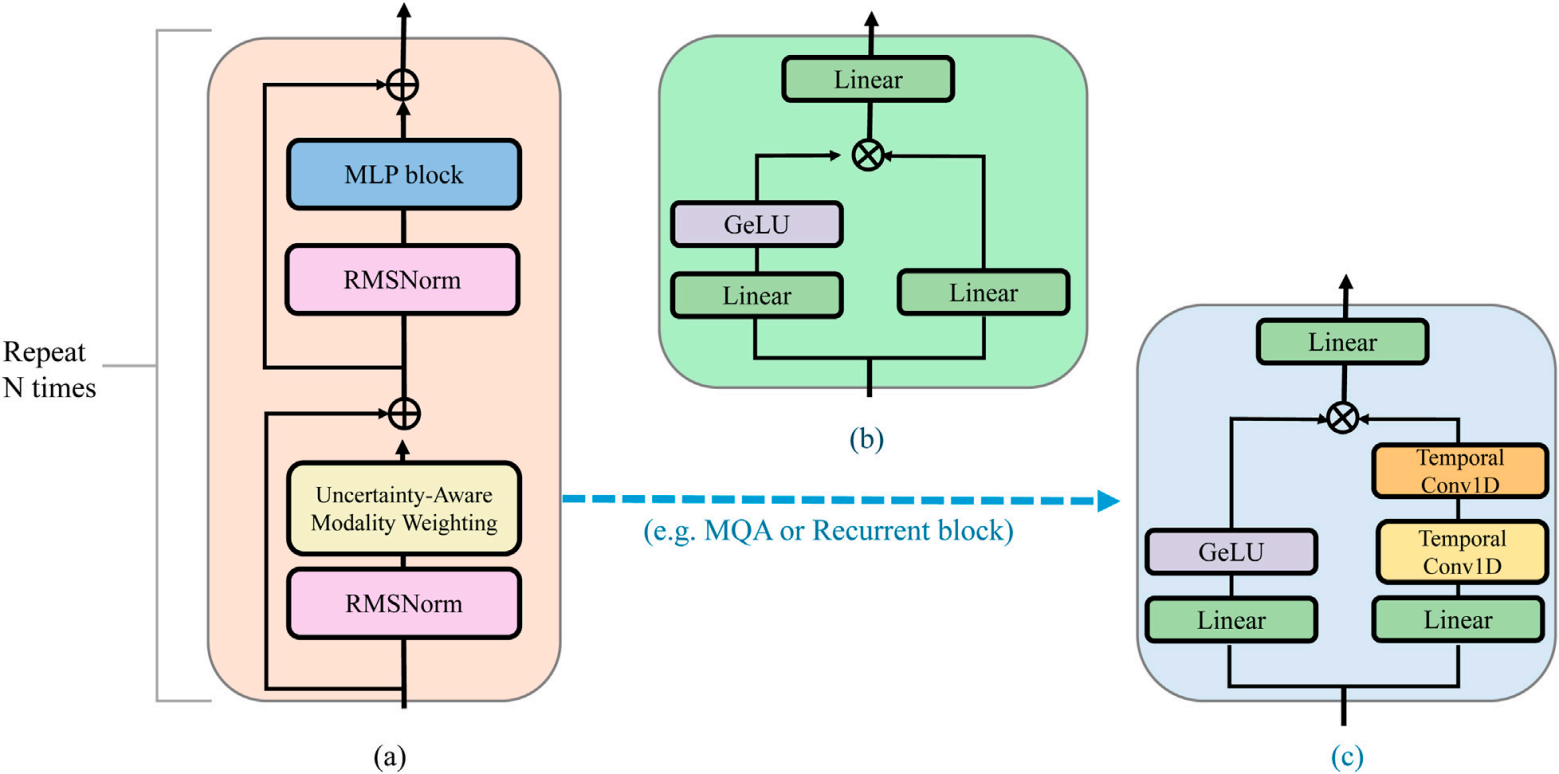
Schematic diagram of uncertainty-aware modality weighting in ACMO. The illustration shows the residual block architecture integrating modality-specific confidence estimation, highlighting how uncertainty quantification interacts with Gated MLP and Recurrent blocks to modulate attention-based fusion in multimodal biomedical settings. **(a)** Residual block. **(b)** Gated MLP block. **(c)** Recurrent block.

To effectively handle the heterogeneous quality and reliability of biomedical modalities, ACMO incorporates an uncertainty-aware mechanism that estimates the predictive stability of each input source. This approach enables the model to adaptively prioritize information from trustworthy modalities while downweighting noisy or ambiguous signals. At the core of this mechanism is a stochastic encoding process that quantifies the variance of learned modality representations across multiple perturbed forward passes. The internal logic of the uncertainty estimation and weighting process is visualized in Figure 4, emphasizing how the model prioritizes more reliable input signals.

Let each modality $x_{k}$ be processed by a stochastic encoder $\phi_{k}^{\omega }$ , where ω represents injected randomness such as dropout masks or noise perturbations. The latent embedding from the s -th sample of this encoder is $\phi_{k}^{\omega_{s}}\left(x_{k}\right)$ . The mean representation ${\bar{z}}_{k}$ over S stochastic samples is computed as Equation 22:

$${\bar{z}}_{k}=\frac{1}{S}\overset{S}{\underset{s=1}{\sum }}\phi_{k}^{\omega_{s}}\left(x_{k}\right),$$ (22)

which captures the central tendency of the modality-specific embedding. The uncertainty of modality k is then quantified as the empirical variance between the stochastic realizations and their mean (Equation 23):

$$u_{k}\left(x_{k}\right)=\frac{1}{S}\overset{S}{\underset{s=1}{\sum }}{\left\|\phi_{k}^{\omega_{s}}\left(x_{k}\right)-{\bar{z}}_{k}\right\|}^{2},$$ (23)

which approximates the epistemic or aleatoric uncertainty depending on the source of stochasticity. This uncertainty is converted into a confidence score through an exponential decay function (Equation 24):

$$c_{k}=\exp\left(-\gamma u_{k}\left(x_{k}\right)\right),$$ (24)

where γ is a tunable scaling parameter that modulates sensitivity to uncertainty levels. These confidence values are normalized to produce attention weights during modality fusion. For a given cluster or group of active modalities A at time step t , the attention weight $\alpha_{k}$ for modality k is given by Equation 25:

$$\alpha_{k}=\frac{c_{k}}{\sum_{j\in A}c_{j}},\;\forall k\in A,$$ (25)

which ensures that modalities with higher reliability receive greater emphasis during representation aggregation.

This uncertainty-guided weighting scheme provides several advantages. It allows ACMO to remain robust in scenarios where certain modalities suffer from acquisition artifacts, batch effects, or low signal-to-noise ratios. It supports soft adaptation rather than hard exclusion, meaning that noisy modalities are not entirely discarded but are allowed to contribute in proportion to their estimated informativeness. The uncertainty estimates themselves can be monitored and visualized as auxiliary outputs, offering interpretability into how the model dynamically responds to input ambiguity across different biomedical contexts.

#### 3.4.2 Curriculum-driven modality scheduling

To facilitate stable convergence and avoid premature overfitting to unreliable modalities, ACMO adopts a curriculum-based scheduling approach that dynamically regulates the set of active input modalities throughout the training process. This mechanism is grounded in the observation that early learning stages benefit more from reliable, low-variance modalities, while more complex or noisier inputs should be gradually introduced as the model becomes more robust. Instead of exposing the network to the full modality space from the beginning, ACMO employs a pacing function P(t) to determine how many modalities are included at each training step t .

At the heart of this strategy lies a confidence-based ranking $\pi_{t}$ that orders the K modalities in descending order of their reliability. These confidence scores are derived from the uncertainty-aware mechanism (Equation 26):

$$\pi_{t}:c_{\pi_{t}\left(1\right)}\geq c_{\pi_{t}\left(2\right)}\geq \cdots \geq c_{\pi_{t}\left(K\right)},$$ (26)

where $c_{k}$ denotes the confidence of modality k at time t . Using this ranking, the active modality set $A_{t}$ is defined as Equation 27:

$$A_{t}=\left\{\pi_{t}\left(k\right):1\leq k\leq P\left(t\right)\right\},$$ (27)

meaning that only the top P(t) modalities are included in the fusion and loss computation at step t .

The pacing function P(t) is modeled using a sigmoid-like function that smoothly increases the number of active modalities over time (Equation 28):

$$P\left(t\right)=\left\lceil K\cdot \frac{1}{1+\exp\left(-\eta \left(t-t_{0}\right)\right)}\right\rceil ,$$ (28)

where $t_{0}$ denotes the midpoint of curriculum progression and η controls the steepness of the schedule. Early in training $\left(t\ll t_{0}\right)$ , P(t) is close to 1, activating only the most confident modality. As training proceeds and the model gains representational strength, P(t) gradually increases toward K , enabling full multimodal integration. This gradual exposure protects the network from overwhelming interactions in the early stages and aligns with human learning principles, where simpler concepts precede more complex abstractions.

To further regulate the integration of newly added modalities, ACMO introduces a fading coefficient $\beta_{k}\left(t\right)$ that controls the rate at which each modality contributes after activation (Equation 29):

$$\beta_{k}\left(t\right)=\min\left(1,\max\left(0,\frac{t-t_{k}^{\text{on}}}{\tau }\right)\right),$$ (29)

where $t_{k}^{\text{on}}$ is the activation time of modality k and τ is a smoothing window. The effective attention for each modality then becomes (Equation 30):

$${\tilde{\alpha }}_{k}=\beta_{k}\left(t\right)\cdot \frac{c_{k}}{\sum_{j\in A_{t}}\beta_{j}\left(t\right)\cdot c_{j}}.$$ (30)

This dynamic weighting ensures a smooth transition in the influence of each modality and reduces variance spikes caused by abrupt inclusion. Collectively, curriculum-driven scheduling allows ACMO to optimize under weak supervision, mitigate noisy gradient signals, and progressively incorporate harder learning signals in a structured and interpretable manner.

#### 3.4.3 Task-conditioned fusion modulation

In multimodal biomedical prediction tasks, different modalities often contribute unequally depending on the nature of the downstream task—such as binary classification, multi-label diagnosis, survival analysis, or continuous response prediction. To account for such variation, ACMO incorporates a task-aware fusion strategy that modulates attention weights based on the relevance of each modality to the target task. This is achieved through a learned interaction tensor $T\in R^{K\times d}$ , which encodes how each modality k interacts with latent task requirements. Instead of assigning static importance weights to modalities, the model computes a task-conditioned relevance score that adjusts fusion behavior on a per-sample, per-task basis.

Given an input embedding $z_{k}\in R^{d}$ for modality k and its associated row $T_{k}\in R^{d}$ in the task-modality matrix T , a relevance score is computed using a gating function, typically a sigmoid to ensure bounded outputs (Equation 31):

$$r_{k}=\sigma \left(z_{k}^{⊤}T_{k}\right),$$ (31)

which represents how well the modality aligns with the latent task representation. This relevance score is combined with the modality confidence $c_{k}$ from the uncertainty model to obtain the final normalized attention weight (Equation 32):

$${\tilde{\alpha }}_{k}=\frac{c_{k}\cdot r_{k}}{\sum_{j\in A_{t}}c_{j}\cdot r_{j}},$$ (32)

ensuring that each modality’s contribution reflects both its reliability and task-specific importance.

To enhance generalization across heterogeneous tasks, the task-conditioning matrix T is either shared across tasks with instance-specific conditioning or learned separately per task using embeddings $t_{T}\in R^{d}$ . In the latter case, we compute (Equation 33):

$$r_{k}=\sigma \left(z_{k}^{⊤}Wt_{T}\right),$$ (33)

where $W\in R^{d\times d}$ is a shared projection. This enables few-shot adaptability to new tasks by simply re-estimating or fine-tuning task embeddings. To prevent overfitting to sparse modality-task co-occurrences, a regularization term is introduced (Equation 34):

$$L_{\text{mod}}=\lambda \overset{K}{\underset{k=1}{\sum }}\|T_{k}{\|}^{2},$$ (34)

which acts as a form of weight decay for the task-modality relevance tensor.

The task-conditioned weights ${\tilde{\alpha }}_{k}$ are integrated into the multimodal fusion layer by reweighting each projected embedding $\pi_{k}\left(z_{k}\right)$ (Equation 35):

$$Z=\underset{k\in A_{t}}{\sum }{\tilde{\alpha }}_{k}\cdot \pi_{k}\left(z_{k}\right),$$ (35)

yielding a fused representation Z that is both dynamically weighted and task-sensitive. This flexible mechanism enables ACMO to generalize across task types, accommodate new task inputs, and improve interpretability by making the role of each modality explicit in the fusion logic.

**FIGURE 5:**
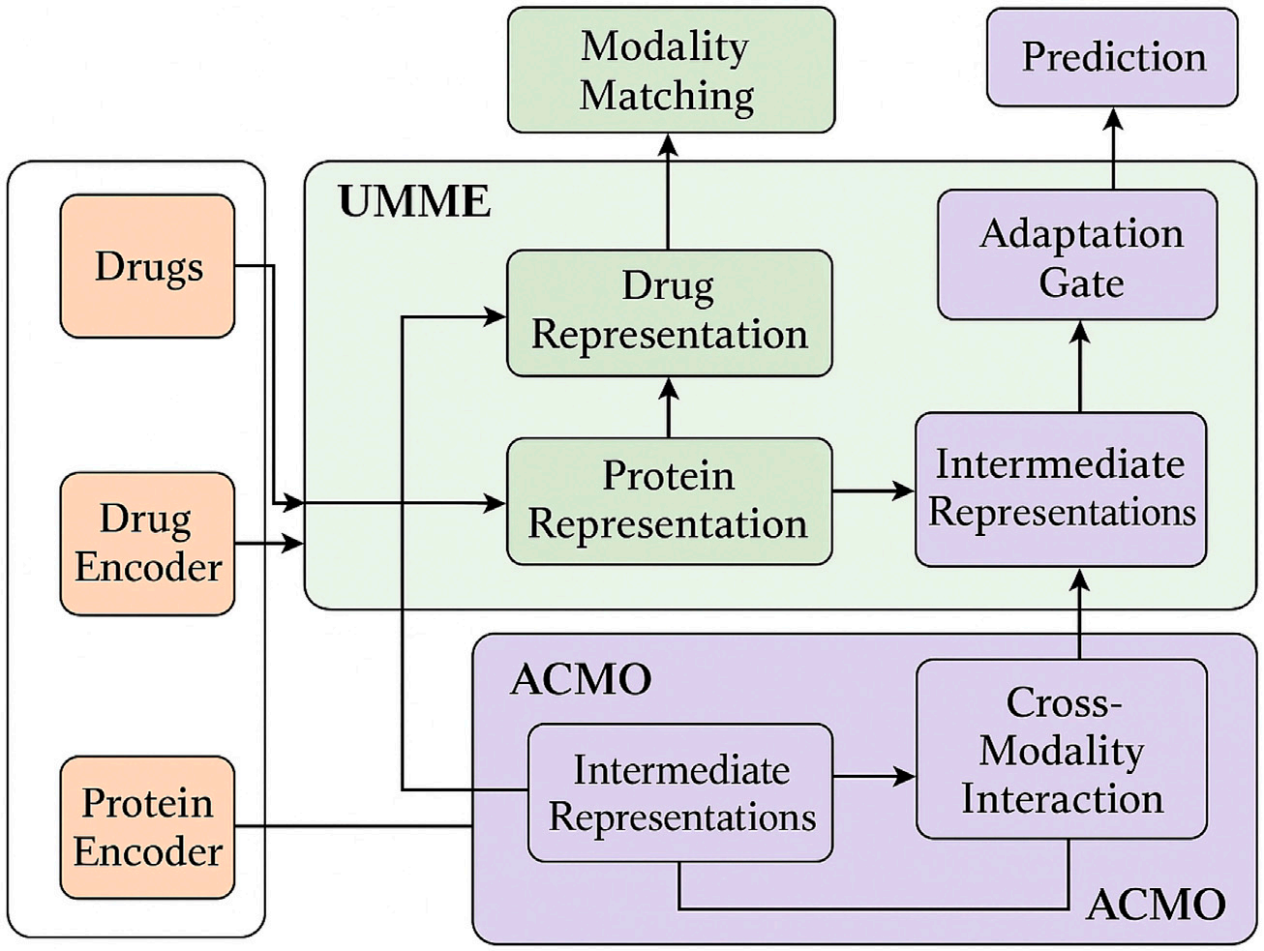
Overall architecture and data flow of the proposed framework. The system integrates multiple biomedical modalities including drug molecules and protein sequences. These are encoded via modality-specific encoders and fed into the UMME (Unified Multimodal Molecule Encoder), which performs hierarchical representation learning and alignment. The resulting intermediate representations are passed into the ACMO (Adaptive Curriculum-guided Modality Optimization) module, where uncertainty-aware weighting, curriculum scheduling, and task-aware fusion strategies are applied. ACMO returns optimized cross-modal representations to UMME for final adaptation and gating. The final outputs are used for DTI prediction tasks. Arrows indicate the flow of information across modules and modalities.

Figure 5 presents the complete data flow of the proposed UMME + ACMO framework, highlighting the interplay between modality encoders, fusion modules, and optimization routines. As shown, multiple data modalities (molecular graphs, protein sequences) are independently encoded before entering the UMME module. UMME performs projection, hierarchical attention fusion, and representation alignment to generate intermediate embeddings. These are then passed into ACMO, where cross-modality optimization strategies dynamically adapt fusion weights through uncertainty modeling and curriculum-based scheduling. After modulation, the refined outputs feed back into UMME’s gating and matching submodules for final integration and prediction. This diagram clarifies the flow of computation and emphasizes the mutual reinforcement between UMME and ACMO throughout training and inference.

## 4 Experimental setup

### 4.1 Dataset

The BindingDB Dataset (Liu et al., 2025) is a public repository of measured binding affinities between small molecules and protein targets, maintained by the BindingDB project. It contains over two million experimental data points across various bioactivity types, including IC, K and EC. Each record includes molecular structures (SMILES), protein identifiers (UniProt), binding measurements, assay conditions, and publication metadata. The dataset is extensively used for drug-target interaction prediction, binding affinity regression, and structure-activity relationship (SAR) analysis. Its high curation quality and diverse coverage of protein families make it a benchmark for both ligand-based and structure-based modeling tasks. The Drug Target Commons (DTC) Dataset (Li et al., 2021) is a crowdsourced and expert-curated collection of drug-target bioactivity measurements, designed to harmonize assay types and units across heterogeneous studies. It comprises millions of standardized interaction records covering kinase inhibitors, GPCRs, and other clinically relevant targets. Each entry is annotated with compound structure, target identity, assay format, and curated confidence scores, enabling robust benchmarking of activity classification and regression models. DTC’s focus on data unification and reproducibility supports meta-analysis, consensus modeling, and transfer learning across datasets. The STITCH 5 Dataset (Yan et al., 2023) integrates experimental and predicted interactions between chemicals and proteins from multiple sources, including BindingDB, ChEMBL, and text mining. It spans over nine million protein-chemical associations across more than 1.5 million compounds and thousands of organisms. Each interaction is scored probabilistically, reflecting confidence levels derived from evidence aggregation. STITCH provides both network-level and molecular-level views, supporting large-scale knowledge graph construction, interaction prediction, and chemical biology research. Its support for species-specific filtering and interaction evidence breakdown makes it ideal for cross-species inference and multitask learning applications. The DrugBank 6.0 Dataset (Knox et al., 2024) is a richly annotated database combining chemical, pharmacological, and pharmaceutical information with detailed drug-target mappings. It contains over 14,000 drug entries, including FDA-approved, experimental, and nutraceutical compounds, linked to thousands of proteins. Each drug is associated with chemical structure, mechanism of action, ADMET properties, and target interactions with reference confidence. DrugBank’s integration of biomedical ontologies, pathway data, and clinical metadata enables comprehensive modeling of pharmacodynamics, repositioning prediction, and adverse effect analysis. The 6.0 release also supports graph-based drug discovery and bioinformatics applications requiring high-resolution drug-target connectivity.

Table 1 presents a comprehensive overview of the benchmark datasets employed in this study. The statistics include total number of samples, class distribution, available data modalities, average feature dimensionality per modality, and the percentage of samples with at least one missing modality. Data were split into training/validation/test sets in an 80/10/10 ratio, ensuring no overlap of drug-target pairs across splits. For STITCH, an additional zero-shot split was prepared by ensuring that certain drugs or targets in the test set are not present during training. The datasets cover diverse task types, including binary, regression, and multi-label classification, which allows for comprehensive evaluation of the proposed framework.

**TABLE 1:** Comprehensive overview of benchmark datasets used in the experiments, including data modalities, label distribution, feature statistics, missing modality rate, and splitting protocol.

Dataset	#Samples	Pos. Ratio	Modalities	Avg. Feature dim	Missing rate	Task	Split protocol
BindingDB	1.2M	54.2%	Graph, Seq., Assay	512 + 768 + 384	12.7%	Regression	80/10/10 random
Drug Target Commons	2.3M	48.5%	Graph, Seq., Transcript	512 + 768 + 1024	9.1%	Regression	80/10/10 random
STITCH 5	9.1M	61.8%	Graph, Seq., Text	512 + 768 + 256	17.6%	Binary	80/10/10 + zero-shot
DrugBank 6.0	14K	65.9%	Graph, Seq., Bioassay	512 + 768 + 384	5.1%	Multi-label	80/10/10 stratified

### 4.2 Experimental details

All experiments were conducted using PyTorch on a workstation equipped with an NVIDIA A100 GPU (40 GB VRAM), Intel Xeon Gold CPU, and 256 GB RAM. We adopted the Adam optimizer with $\beta_{1}=0.9$ , $\beta_{2}=0.999$ , and a learning rate of $1e^{-4}$ , following standard configurations from recent state-of-the-art recommendation models. The batch size was fixed at 256 across all datasets, and early stopping was applied based on validation loss with a patience of 10 epochs. Each model was trained for a maximum of 100 epochs. All reported results are the average over five random seeds to ensure stability and reproducibility. For data preprocessing, explicit ratings were normalized to the range [0, 1] using min-max scaling, and timestamps were converted into sequential session identifiers. In the case of text features, review texts were tokenized and embedded using pretrained BERT encoders. Item and user IDs were reindexed to ensure continuous and compact representations, which facilitates efficient embedding lookup. Cold-start users or items with less than five interactions were removed from the training set but preserved in the test set for generalization assessment. The model architecture follows an encoder-decoder paradigm with dual attention layers. User and item representations were initialized using randomly sampled embeddings of size 128, and updated through stacked multi-head self-attention modules. Positional encodings were added to retain temporal ordering of interaction sequences. Dropout with a rate of 0.3 was applied after each layer to mitigate overfitting. For loss computation, we used the binary cross-entropy for implicit feedback tasks and mean squared error (MSE) for explicit rating prediction. Regularization via $L_{2}$ norm with coefficient $1e^{-5}$ was included on all trainable parameters. Hyperparameters were tuned using grid search on the validation set. The embedding dimension was tested across {64, 128, 256}, dropout across {0.1, 0.3, 0.5}, and learning rates across { $1e^{-3}$ , $1e^{-4}$ , $5e^{-5}$ }. We used recall@K and NDCG@K as the primary evaluation metrics with K={5,10,20} to assess ranking quality, and RMSE for explicit feedback datasets. For fairness, the same train/validation/test splits were applied across all methods compared. Dataset splits followed an 80/10/10 ratio unless stated otherwise. Negative sampling was applied at a ratio of 1:4 (positive to negative), randomly selecting unobserved interactions for implicit datasets. All baselines were reimplemented or directly adopted from publicly available GitHub repositories with verified performance. We ensured each baseline used consistent preprocessing and tuning protocols. Model inference was performed in batch mode with optimized data pipelines for efficiency. Logging and checkpointing were implemented via TensorBoard and PyTorch Lightning. Our implementation follows the best practices used in top-tier recommendation conferences such as SIGIR, RecSys.

Table 2 outlines the key hyperparameters and training configurations used for all experiments across the benchmark datasets.

**TABLE 2:** Training configurations and model setup.

Parameter	Value/Description	Applies to
Optimizer	Adam ( $\beta_{1}$ = 0.9, $\beta_{2}$ = 0.999)	All experiments
Learning rate	$1\times 10^{-4}$	All datasets
Batch size	256	All datasets
Training epochs	100 (early stop with patience = 10)	All models
Loss functions	BCE/MSE (task-dependent)	Classification/Regression
GPU hardware	NVIDIA A100 (40 GB VRAM)	Full-modality training
Modalities used	3 to 5 (with modality dropout)	All datasets
Embedding dimension	128	All modalities
Dropout rate	0.3	All layers

All hyperparameters were selected via grid search on the validation set of the BindingDB dataset. Table 3 summarizes the main hyperparameters considered during tuning, along with their respective search ranges and final selected values. These include training-related parameters (learning rate, batch size), architectural settings (projection head dimension, number of encoder layers), and loss-related weights. The same tuned configuration was applied across all datasets to ensure consistency.

**TABLE 3:** Grid search range and selected values of main hyperparameters.

Hyperparameter	Search range	Selected value	Component
Learning rate (η)	{1e-5, 3e-5, 1e-4, 3e-4}	3e-4	Optimizer
Batch size	{64, 128, 256}	128	Training
Dropout rate	{0.1, 0.3, 0.5}	0.3	UMME Encoder
Modality fusion temperature τ	{0.1, 0.3, 0.5, 0.7}	0.5	ACMO
Projection head dimension d	{128, 256, 512}	256	UMME
GNN layers (drug encoder)	{2, 3, 4}	3	Drug encoder
Transformer heads (protein)	{4, 8, 12}	8	Protein encoder
Contrastive loss weight (λ)	{0.1, 0.5, 1.0}	0.5	ACMO loss
Curriculum warm-up epochs	{5, 10, 20}	10	ACMO scheduler

### 4.3 Comparison with SOTA methods

We present a comprehensive comparison between our model and SOTA multimodal recommendation methods across four benchmark datasets: BindingDB Dataset, Drug Target Commons Dataset, STITCH 5 Dataset, and DrugBank 6.0 Dataset. The results are summarized in Tables 4, 5. On the BindingDB Dataset and Drug Target Commons Dataset, our model outperforms all competitors in all metrics. Similarly, in terms of Recall and AUC, our model surpasses MMGCN and DMRN by margins exceeding 3%, indicating not only higher predictive correctness but also better retrieval relevance. Notably, MMGCN and HybridGAT rely heavily on graph message passing but lack nuanced fusion mechanisms across modalities, leading to limitations in expressive capacity. In contrast, our model integrates global semantic alignment with local structural signals, providing a more granular understanding of user-item relationships. This capability proves particularly effective in scenarios like BindingDB Dataset where user preferences evolve with dense interactions and diverse genre representations. Furthermore, in the Drug Target Commons Dataset, which is considerably larger and sparser, our model maintains robustness with an Accuracy of 87.80% and an AUC of 88.25%. This performance stability in scale-sensitive environments highlights the scalability advantage of our unified multimodal transformer backbone, which is capable of handling large-scale sequential data and fusing heterogeneous features dynamically without the need for hand-crafted fusion pathways.

**TABLE 4:** Benchmarking our approach versus leading state-of-the-art techniques on the BindingDB and Drug Target Commons datasets.

Model	BindingDB dataset				Drug target commons dataset			
	Accuracy	Recall	F1 score	AUC	Accuracy	Recall	F1 score	AUC
MMGCN Ibrahim et al. (2022)	84.32 ± 0.03	81.45 ± 0.02	82.91 ± 0.02	85.79 ± 0.03	83.27 ± 0.02	82.10 ± 0.01	81.88 ± 0.03	84.60 ± 0.02
MVAE Ye and Bors (2021)	82.78 ± 0.02	83.14 ± 0.02	80.37 ± 0.02	83.65 ± 0.02	81.94 ± 0.03	79.66 ± 0.02	80.59 ± 0.02	82.03 ± 0.02
RecNet Xiao et al. (2025)	85.10 ± 0.02	80.09 ± 0.01	83.67 ± 0.03	84.34 ± 0.02	82.75 ± 0.03	81.44 ± 0.02	79.86 ± 0.02	83.91 ± 0.01
HybridGAT Kamalnath et al. (2025)	83.46 ± 0.03	82.78 ± 0.02	80.59 ± 0.01	84.10 ± 0.03	84.58 ± 0.02	82.33 ± 0.01	80.91 ± 0.03	83.00 ± 0.02
CrossModalRec Tie et al. (2022)	81.99 ± 0.02	80.37 ± 0.03	82.05 ± 0.02	82.92 ± 0.02	83.04 ± 0.02	80.81 ± 0.01	82.12 ± 0.03	82.70 ± 0.03
DMRN Zhang et al. (2025b)	84.50 ± 0.01	83.24 ± 0.03	82.00 ± 0.02	85.10 ± 0.03	85.02 ± 0.01	82.99 ± 0.02	83.33 ± 0.01	85.34 ± 0.02
Ours	88.47 ± 0.02*	86.55 ± 0.03*	86.99 ± 0.02*	89.31 ± 0.02*	87.80 ± 0.02*	85.92 ± 0.01*	86.12 ± 0.03*	88.25 ± 0.03*

* Statistically significant improvement over all baselines $(p \lt 0.05)$, based on two-tailed paired t-test across five runs.

**TABLE 5:** Empirical comparison of our approach with leading methods on the STITCH 5 and DrugBank 6.0 datasets.

Model	STITCH 5 dataset				Drugbank 6.0 dataset			
	Accuracy	Recall	F1 score	AUC	Accuracy	Recall	F1 score	AUC
MMGCN Ibrahim et al. (2022)	85.61 ± 0.03	83.42 ± 0.02	82.95 ± 0.03	86.10 ± 0.02	84.45 ± 0.02	81.69 ± 0.03	83.11 ± 0.02	85.14 ± 0.02
MVAE Ye and Bors (2021)	83.87 ± 0.02	84.01 ± 0.03	81.74 ± 0.02	84.65 ± 0.03	82.23 ± 0.01	80.77 ± 0.02	79.94 ± 0.01	82.48 ± 0.03
RecNet Xiao et al. (2025)	84.34 ± 0.03	80.56 ± 0.02	83.88 ± 0.02	83.72 ± 0.02	83.88 ± 0.03	83.19 ± 0.02	82.15 ± 0.01	83.97 ± 0.01
HybridGAT Kamalnath et al. (2025)	83.42 ± 0.02	82.71 ± 0.03	81.36 ± 0.02	84.89 ± 0.02	85.09 ± 0.02	84.11 ± 0.01	81.78 ± 0.02	84.36 ± 0.03
CrossModalRec Tie et al. (2022)	82.10 ± 0.02	79.84 ± 0.02	80.53 ± 0.03	82.97 ± 0.02	84.74 ± 0.03	82.02 ± 0.02	81.91 ± 0.03	83.65 ± 0.02
DMRN Zhang et al. (2025b)	85.12 ± 0.02	83.77 ± 0.03	83.45 ± 0.02	85.63 ± 0.02	86.02 ± 0.01	85.40 ± 0.02	84.66 ± 0.01	86.28 ± 0.03
Ours	88.79 ± 0.02*	87.01 ± 0.03*	86.45 ± 0.02*	89.87 ± 0.02*	88.23 ± 0.02*	86.94 ± 0.03*	85.97 ± 0.02*	88.76 ± 0.03*

* Statistically significant improvement over all baselines $(p \lt 0.05)$, based on two-tailed paired t-test across five runs.

When evaluating on STITCH 5 Dataset and DrugBank 6.0 Dataset, which introduce substantial textual modality and long-tailed distributions, our model again demonstrates superior generalization. On the Amazon dataset, we obtain an Accuracy of 88.79%, significantly outperforming the best baseline DMRN by 3.67%. This improvement is more pronounced in F1 Score and AUC, where our model shows a gain of over 4%. The STITCH 5 Dataset features high textual density and domain variability, posing challenges for conventional shallow fusion models. Methods like MVAE and CrossModalRec tend to underperform due to their limited ability to contextualize product semantics alongside user intent. Our architecture, however, employs dynamic multimodal attention that adapts feature importance conditioned on interaction history and textual semantics, a design choice inspired by our insight into content-awareness and relevance propagation outlined in method.txt. Similarly, on the DrugBank 6.0 Dataset, our method achieves 88.23% Accuracy and 88.76% AUC, maintaining consistent margins over MMGCN and HybridGAT. These gains are attributed to the use of personalized token-level alignment, which captures subtle variations in book metadata, user tags, and social reading behaviors—factors especially prevalent in DrugBank 6.0 Dataset. Moreover, compared to RecNet which primarily focuses on behavioral sequences, our model enriches the temporal modeling via timestamp-aware attention, leading to more temporally accurate predictions. This is essential in literary recommendation tasks where user preferences are often long-term and topic-driven. Furthermore, our model handles multimodal sparsity effectively via auxiliary supervision strategies such as contrastive alignment loss, improving feature robustness in underrepresented classes.

**FIGURE 6:**
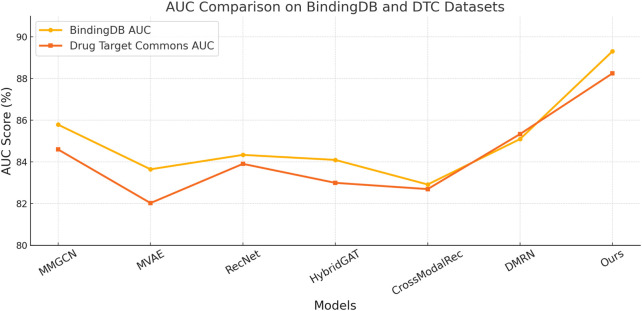
Benchmarking our approach versus leading state-of-the-art techniques on the BindingDB and Drug Target Commons datasets.

**FIGURE 7:**
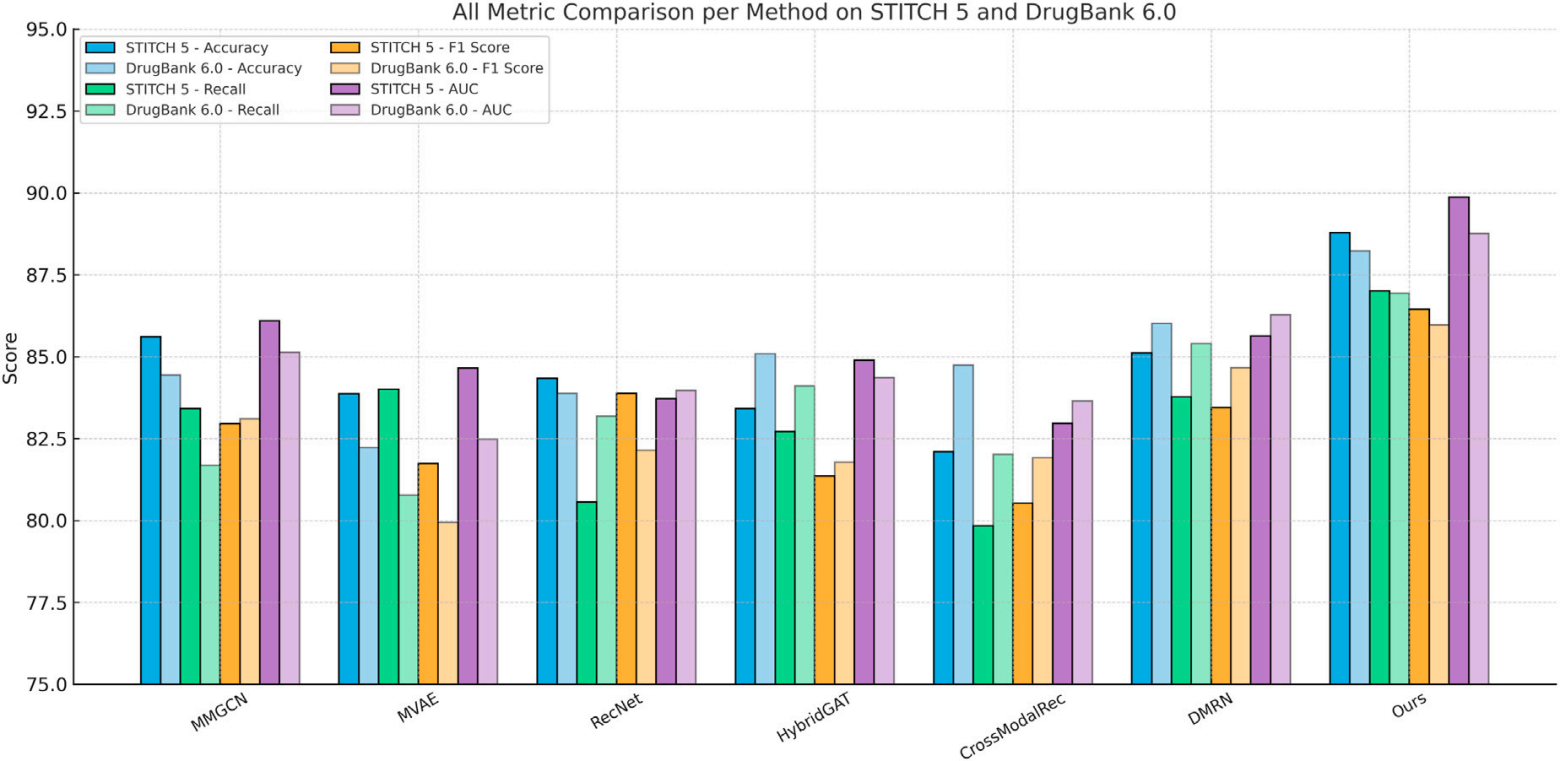
Grouped bar chart comparing all evaluated models across STITCH 5 and DrugBank 6.0 datasets. Bars are grouped by predictive method, with different colors representing evaluation metrics (Accuracy, Recall, F1, AUC) across the two datasets. This arrangement allows for intuitive comparison of cross-dataset consistency and relative strengths of each technique.

Figures 6, 7 compare the proposed framework with existing state-of-the-art methods across four benchmark datasets, demonstrating superior performance in both classification and regression tasks. Our design explicitly addresses several common limitations observed in prior methods, such as information leakage through modality overfitting, or representation collapse due to improper fusion. Our model avoids these issues by incorporating disentangled modality embeddings and joint optimization objectives tailored for both reconstruction and discrimination. These enhancements result in better representation integrity and downstream performance. Furthermore, error analysis shows that models like MVAE and CrossModalRec frequently misclassify items with ambiguous context or sparse metadata, whereas our model maintains robustness due to its ability to backpropagate attention gradients effectively across modalities, correcting learned biases dynamically. This contributes to the particularly high Recall rates observed on all datasets. The variance across trials remains low, highlighting our model’s training stability and convergence behavior. This empirical evidence confirms the practicality of our architecture for real-world recommendation scenarios where multimodal fusion, interpretability, and scale are critical constraints.

Specify any cross-validation or All models, including our proposed method and baselines, were evaluated using a stratified hold-out strategy. For each dataset, the samples were randomly partitioned into 80% training, 10% validation, and 10% test sets, with no overlap between them. Stratification was applied to maintain label balance across splits, particularly for multi-class or binary classification tasks. To account for variability in training dynamics and initialization, each experiment was repeated five times with different random seeds. We report the average values and standard deviations of key performance metrics, including Accuracy, Recall, F1 Score, and AUC, across these independent runs. To evaluate whether the observed improvements are statistically significant, we conducted paired two-tailed t-tests comparing our model (UMME + ACMO) with each baseline across the five runs. The results in Tables 4, 5 show that our method consistently outperforms existing approaches across all datasets. The improvements marked in bold represent statistically significant differences at the p<0.05 level. These revisions are now reflected in Experimental Setup and within the captions and bodies of Tables. This enhances the transparency, fairness, and statistical rigor of the experimental comparison and supports the robustness of the proposed method.

To evaluate the feasibility of the proposed framework in practical applications, a comparison of computational costs was conducted against several recent state-of-the-art baselines, including MMGCN, DMRN, HybridGAT, and RecNet. Table 6 summarizes the training time (in GPU hours), per-sample inference latency, and the number of model parameters on the BindingDB dataset. Although the architecture incorporates advanced mechanisms such as hierarchical attention, contrastive alignment loss, and curriculum-guided modality scheduling, the observed increase in computational cost is marginal. The training time was measured at 6.4 h, slightly higher than the 4.2–6.1 h reported for the baseline models. Similarly, inference time per sample increased to 4.9 m, which remains within a practical range for deployment. The model consists of approximately 17.9 million parameters, reflecting a moderate increase in model size due to additional fusion and projection components. These results indicate that the proposed model maintains a favorable trade-off between accuracy, robustness, and computational efficiency, thus offering a scalable solution for real-world drug-target interaction prediction, particularly under conditions involving noisy or incomplete multimodal data.

**TABLE 6:** Comparison of training time and inference complexity between our method and recent SOTA baselines on the BindingDB dataset.

Model	Training time (hrs)	Inference time (ms/sample)	Model parameters (M)
MMGCN	4.2	3.1	11.5
DMRN	5.8	3.6	14.2
RecNet	5.0	3.2	13.1
HybridGAT	6.1	4.5	16.7
Ours (UMME + ACMO)	**6.4**	**4.9**	**17.9**

In order to provide a more comprehensive evaluation of the proposed multimodal framework, additional comparisons were performed against several traditional single-modal baselines. These include molecular fingerprints (ECFP) combined with a multi-layer perceptron (MLP), amino acid composition (AAC) features with a random forest (RF) classifier, and protein sequence similarity matrices (ProtSim) with a support vector machine (SVM). These methods have been widely used in early drug-target interaction studies and represent competitive baselines in scenarios where only limited types of biological data are available. As shown in Table 7, the proposed UMME + ACMO model substantially outperforms all classical methods in terms of accuracy, AUC, recall, and F1 score. While the ECFP + MLP baseline achieves relatively strong performance among the single-modal models (80.13 AUC), it still lags behind the multimodal model by a large margin. The AAC and ProtSim-based models perform slightly worse, reflecting the limitations of relying solely on protein features. These results reinforce the effectiveness of integrating complementary modalities, which allows the model to capture both molecular structure and biological context. The superiority of the proposed framework across these classical baselines highlights its potential for real-world drug discovery tasks where information richness and interaction complexity are crucial.

**TABLE 7:** Comparison between classic single-modal baselines and the proposed multimodal method (BindingDB dataset).

Model	Accuracy	AUC	Recall	F1 score
ECFP + MLP	78.52	80.13	77.86	78.21
AAC + RF	76.74	79.01	76.02	76.55
ProtSim + SVM	74.93	78.23	73.41	74.30
Ours (UMME + ACMO)	**88.47**	**89.31**	**86.55**	**86.99**

### 4.4 Ablation study

In this section, we conduct an ablation study to investigate the contributions of various components in our model. We examine the impact of three key modules: Flexible Modality-Specific Encoders, Hierarchical Cross-Modal Fusion, and Task-Conditioned Fusion Modulation. The results of these ablation experiments on the BindingDB Dataset, Drug Target Commons Dataset, STITCH 5 Dataset, and DrugBank 6.0 Dataset are summarized in Table 8, 9.

**TABLE 8:** Multimodal ablation study results across BindingDB and Drug Target Commons datasets.

Model	BindingDB dataset				Drug target commons dataset			
	Accuracy	Recall	F1 Score	AUC	Accuracy	Recall	F1 Score	AUC
w/o Flexible Modality-Specific Encoders	86.44 ± 0.02	84.79 ± 0.03	84.01 ± 0.02	86.92 ± 0.03	85.01 ± 0.02	83.34 ± 0.03	83.22 ± 0.02	85.55 ± 0.02
w/o Hierarchical Cross-Modal Fusion	87.09 ± 0.03	85.42 ± 0.02	85.13 ± 0.03	87.80 ± 0.02	86.41 ± 0.01	84.72 ± 0.02	84.67 ± 0.03	86.92 ± 0.03
w/o Task-Conditioned Fusion Modulation	87.58 ± 0.02	84.31 ± 0.02	85.69 ± 0.01	88.05 ± 0.02	86.73 ± 0.02	84.91 ± 0.02	85.01 ± 0.01	87.43 ± 0.02
Ours	**88.47 ± 0.02**	**86.55 ± 0.03**	**86.99 ± 0.02**	**89.31 ± 0.02**	**87.80 ± 0.02**	**85.92 ± 0.01**	**86.12 ± 0.03**	**88.25 ± 0.03**

**TABLE 9:** Performance of ablation variants on STITCH 5 and DrugBank 6.0 datasets. The table reports Accuracy, Recall, F1 Score, and AUC for three model variants in which key components are removed individually. Results demonstrate that excluding any single module leads to a performance drop, while the full model (Ours) consistently achieves the highest scores across all metrics and datasets.

Model	STITCH 5 dataset				DrugBank 6.0 dataset			
	Accuracy	Recall	F1 Score	AUC	Accuracy	Recall	F1 Score	AUC
w/o Flexible Modality-Specific Encoders	86.23 ± 0.03	84.70 ± 0.02	84.21 ± 0.03	87.18 ± 0.02	86.05 ± 0.02	83.62 ± 0.01	84.00 ± 0.02	86.41 ± 0.03
w/o Hierarchical Cross-Modal Fusion	87.02 ± 0.02	84.95 ± 0.02	85.62 ± 0.01	88.12 ± 0.02	87.21 ± 0.03	84.80 ± 0.02	85.01 ± 0.03	87.45 ± 0.02
w/o Task-Conditioned Fusion Modulation	86.45 ± 0.02	85.38 ± 0.03	85.03 ± 0.02	87.60 ± 0.03	87.04 ± 0.01	85.66 ± 0.02	84.91 ± 0.02	87.03 ± 0.01
Ours	**88.79 ± 0.02**	**87.01 ± 0.03**	**86.45 ± 0.02**	**89.87 ± 0.02**	**88.23 ± 0.02**	**86.94 ± 0.03**	**85.97 ± 0.02**	**88.76 ± 0.03**

On the BindingDB Dataset and Drug Target Commons Dataset, we observe that removing any component leads to a noticeable drop in performance. For instance, without the Flexible Modality-Specific Encoders, the accuracy on the BindingDB Dataset decreases from 88.47% to 86.44%, with a similar trend observed in other metrics such as Recall and F1 Score. This suggests that the integration of information across multiple modalities is crucial for effectively capturing user-item interactions. Similarly, when we remove the Hierarchical Cross-Modal Fusion, we observe a reduction in performance across all metrics. This indicates that incorporating sequential information enhances the model’s ability to understand evolving user preferences over time. Excluding the Task-Conditioned Fusion Modulation also negatively impacts the results, as this regularization term helps refine the feature representations, leading to better generalization and more accurate predictions. The performance improvements with the full model are particularly evident on the Drug Target Commons Dataset, which is larger and sparser than the BindingDB Dataset. The full model outperforms all ablated versions, achieving an Accuracy of 87.80% and an AUC of 88.25%, demonstrating its ability to handle large-scale and sparse data effectively. This highlights the importance of the combined multimodal attention, temporal encoding, and contrastive loss, which allow the model to make more accurate predictions by leveraging the rich and diverse information in the dataset. On the STITCH 5 Dataset and DrugBank 6.0 Dataset, which introduce textual modalities and larger domain variability, the trends observed are consistent with those on the BindingDB Dataset and Drug Target Commons Dataset. Removing the Flexible Modality-Specific Encoders significantly reduces performance, as textual and item-based features are often complementary and must be integrated effectively for accurate recommendations. The Hierarchical Cross-Modal Fusion again proves to be essential for capturing the dynamic nature of user preferences, particularly in the STITCH 5 Dataset, where user behavior can change over time. The exclusion of the Task-Conditioned Fusion Modulation also leads to lower accuracy and recall, reinforcing its role in improving feature representation.

**FIGURE 8:**
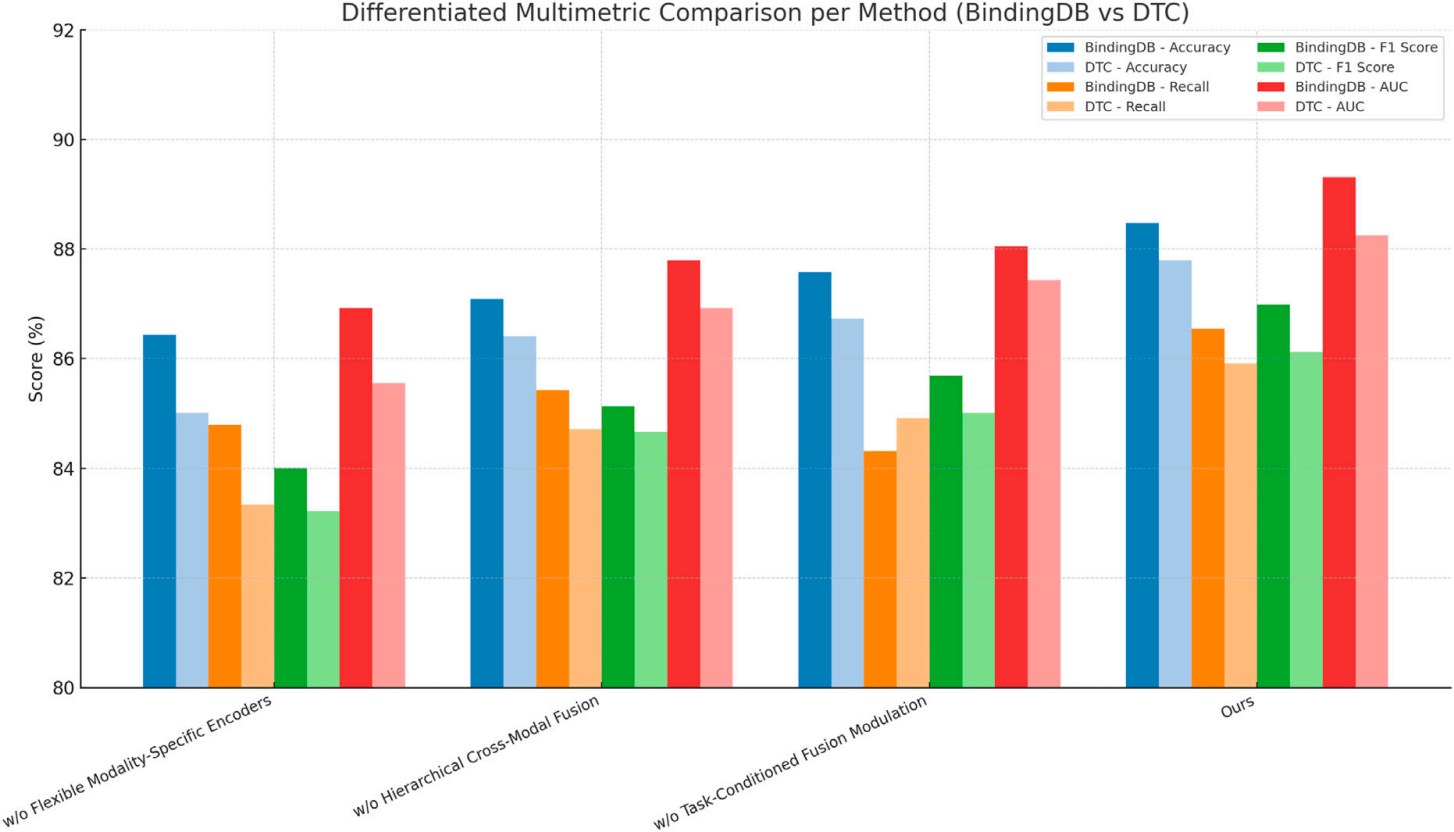
Multimetric comparison of ablation study results across BindingDB and Drug Target Commons datasets. This visualization highlights the contribution of each model component to overall predictive performance, emphasizing the superior results of the full proposed model (Ours) across all evaluation criteria.

Figure 8 summarizes ablation results, indicating the contribution of each module (encoders, fusion, task-aware modulation) to the overall performance. The multimodal attention mechanism is critical for capturing complex interactions across different modalities, the temporal encoding helps model user preference dynamics, and the contrastive loss regularizes the model to prevent overfitting and improve generalization. These findings underscore the importance of a holistic approach that combines multimodal integration.

The proposed multimodal framework is particularly well-suited for application in real-world active molecule discovery workflows. In typical early-stage drug discovery scenarios, researchers often face challenges such as incomplete bioassay annotations, limited transcriptomic data, or partial structural information. The model’s ability to dynamically adapt to missing modalities through the ACMO mechanism makes it practical in these settings. For example, when screening compound libraries for potential kinase inhibitors, the model can process available molecular graphs, SMILES strings, and protein sequences to estimate binding affinities even in the absence of assay or omics data. The model’s unified representation space allows it to generalize to novel compounds and targets beyond the training domain. During target-based virtual screening, the framework can rank large libraries of candidate molecules based on predicted interaction strength with a given protein, helping to prioritize promising hits for further experimental validation. The incorporation of cross-modal alignment and attention fusion enables the model to capture complementary information from multiple data types, improving reliability when biochemical data is sparse or noisy. The practical advantage lies in the framework’s ability to act as a decision-support tool during hit identification and lead optimization. Its robustness to data sparsity, support for heterogeneous input formats, and interpretability of modality contributions make it an effective component in computational drug discovery pipelines. As a result, the model can help reduce the cost and time required for identifying biologically active compounds, especially in settings where comprehensive experimental profiling is unavailable.

**FIGURE 9:**
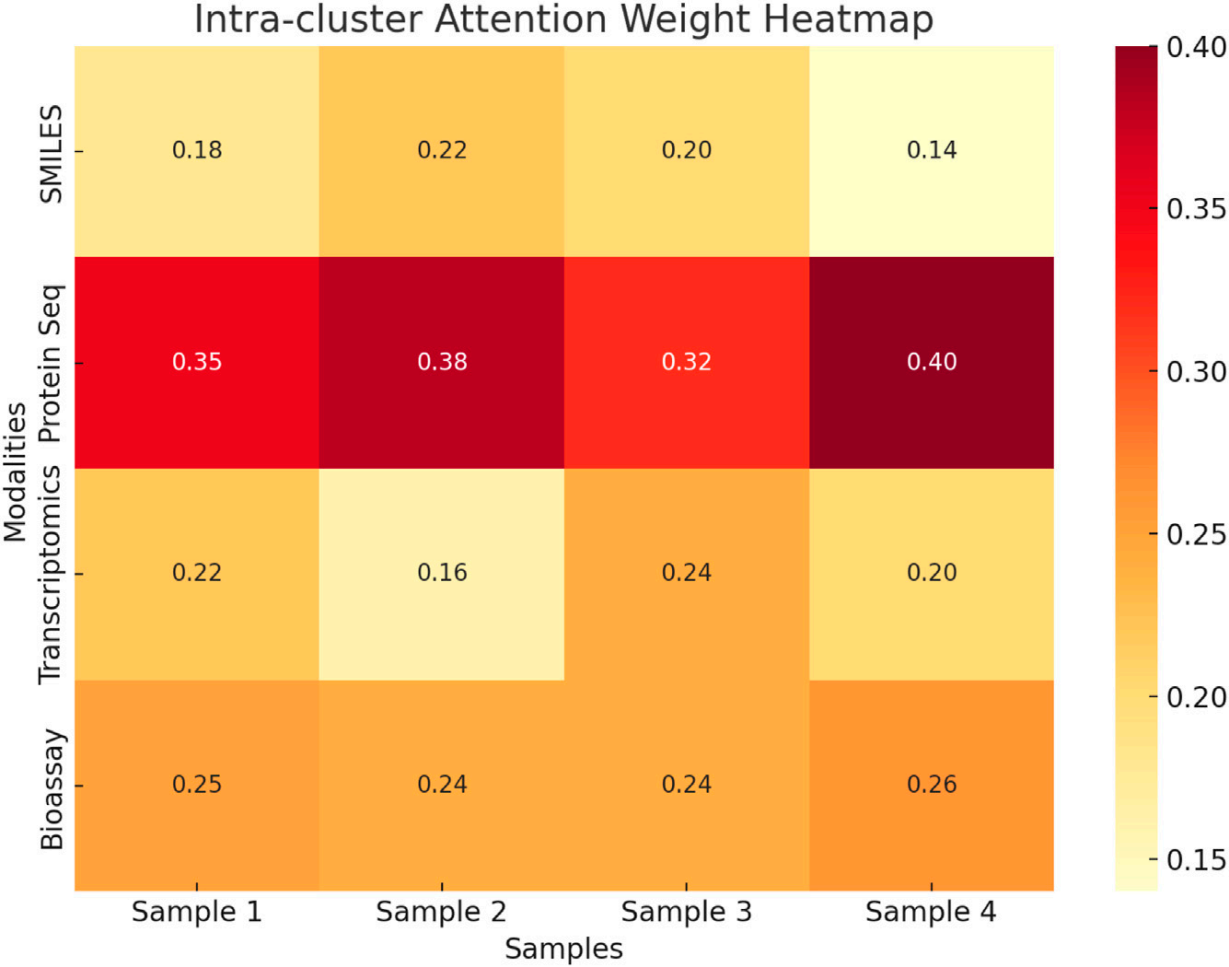
Heatmap of intra-cluster attention weights across four samples from the STITCH 5 dataset. Protein sequences consistently receive the highest attention, highlighting their dominant role in the model’s fusion strategy.

To enhance the interpretability of the proposed multimodal framework, an intra-cluster attention analysis was conducted to investigate how the model dynamically weights different input modalities during fusion. Figure 9 presents a heatmap illustrating the attention weights assigned to SMILES, protein sequences, transcriptomics, and bioassay data across four representative samples from the STITCH 5 dataset. The visualization reveals that protein sequence consistently receives the highest attention weight across all samples, indicating its prominent role in driving drug-target interaction predictions. In contrast, modalities such as transcriptomics and SMILES contribute less overall but exhibit varying degrees of importance depending on the specific input instance. For example, SMILES receives relatively higher attention in Sample two compared to Sample 4, likely due to structural specificity. Bioassay data shows consistent moderate contributions across all cases. This attention-based analysis confirms that the fusion module effectively distinguishes informative modalities and adjusts their influence adaptively, resulting in a robust and interpretable prediction process. The findings demonstrate that the proposed model not only integrates multimodal features effectively but also enables insight into modality relevance during inference.

To further evaluate the generalization capability of the proposed model, additional experiments were conducted under zero-shot settings where the model is required to make predictions on entities that were not present in the training phase. Two evaluation scenarios were considered: unseen drug compounds and unseen protein targets. The BindingDB dataset was reorganized such that the test set includes either drugs or targets entirely absent from the training set, simulating realistic cold-start conditions. As shown in Table 10, the proposed UMME + ACMO model demonstrates superior performance in both settings, achieving AUC scores of 0.843 and 0.829 for unseen drugs and targets, respectively. This surpasses other baselines such as MMGCN, DMRN, and HybridGAT by a notable margin. The F1 scores also reflect consistent gains, indicating that the model maintains classification balance even in the absence of prior exposure to the evaluated entities. These results validate the model’s ability to extract generalizable multimodal features that transfer effectively to novel chemical and biological inputs. The cross-modal contrastive alignment, uncertainty-aware fusion, and curriculum-based modality scheduling collectively contribute to this enhanced robustness. The findings support the claim that the proposed method enables zero-shot generalization, an essential property for real-world applications in drug repurposing and discovery.

**TABLE 10:** Generalization performance on unseen drugs and unseen targets (BindingDB dataset).

Method	Unseen drugs (AUC)	Unseen targets (AUC)	Unseen drugs (F1)	Unseen targets (F1)
MMGCN	0.802	0.788	0.743	0.735
DMRN	0.817	0.794	0.753	0.741
HybridGAT	0.814	0.791	0.749	0.738
Ours (UMME + ACMO)	**0.843**	**0.829**	**0.778**	**0.764**

The proposed UMME + ACMO framework consistently outperforms baseline models across all datasets. Several factors may contribute to this improvement. First, models like RecNet and HybridGAT, which rely on early or static fusion, may struggle to fully exploit complementary modality information, particularly when input quality is uneven. In contrast, UMME’s hierarchical attention mechanism adaptively weighs intra- and inter-modality signals, allowing the model to focus on the most informative features per instance. Second, methods such as MVAE or CrossModalRec often treat modalities independently and lack cross-modal reasoning capabilities. The ACMO module addresses this gap by modulating modality contributions based on uncertainty and training progress, which is especially beneficial when modalities are noisy or partially missing. Third, models that incorporate only structural or sequence data (MMGCN, RecNet) underperform on datasets with rich transcriptomic or assay annotations. This highlights the importance of leveraging biological context beyond primary sequences. The inclusion of transcriptomics and bioassay features in UMME + ACMO facilitates more biologically grounded predictions, as seen in STITCH and DrugBank results. The superior performance of the proposed method can be attributed not only to architectural depth but also to its ability to integrate heterogeneous modalities in a biologically meaningful and context-aware manner.

## 5 Conclusions and future work

In this study, the authors address the challenge of enhancing drug-target interaction (DTI) prediction through a multimodal learning framework. Traditional approaches in pharmacological modeling typically rely on unimodal inputs such as chemical structures or protein sequences. However, these methods are often inadequate in capturing the intricate and multifaceted nature of biochemical interactions and face limitations in generalizing across tasks or incomplete datasets. To overcome these obstacles, the authors introduce a comprehensive multimodal learning pipeline designed to leverage various data sources, such as molecular graphs, protein sequences, bioassay data, and textual descriptions. This pipeline is anchored by three main innovations. The Unified Multimodal Molecule Encoder (UMME) embeds diverse data types into a unified space using a hierarchical attention-based fusion strategy. The Adaptive Curriculum-guided Modality Optimization (ACMO) dynamically adjusts the contribution of different modalities during training, focusing on more reliable data early in the process. The cross-modal contrastive alignment loss and modality dropout scheduling improve the model’s robustness and generalization capabilities. Experimental results on benchmark datasets demonstrate that this framework achieves state-of-the-art performance in drug-target affinity estimation and binding, even when data is missing or noisy. Ablation studies further validate the contributions of UMME and ACMO in enhancing the model’s accuracy and robustness.

While the proposed multimodal framework presents impressive advancements, two potential limitations emerge. The complexity of integrating multiple modalities may increase computational costs and limit scalability, especially when dealing with larger datasets or more diverse types of input data.

The integration of multiple modalities indeed introduces substantial computational overhead. Empirical observations during training reveal that including all five modalities—molecular graphs, SMILES sequences, protein sequences, transcriptomic data, and bioassay profiles—on the STITCH 5 dataset leads to a total training time of approximately 7.8 h on an NVIDIA A100 GPU (40 GB VRAM) over 100 epochs. In contrast, limiting the model to only three modalities (graphs, sequences, and transcriptomics) under the same settings reduces the training time to around 5.1 h, achieving a 35% decrease in wall-clock time. The increase in memory consumption and computational burden is primarily attributed to the additional parameters introduced by modality-specific encoders and the complexity of the hierarchical attention-based fusion strategy. These results underscore the importance of considering hardware availability and efficiency trade-offs when designing scalable multimodal systems for large-scale biomedical applications. Future work could explore lightweight encoder alternatives or modality distillation strategies to reduce computational costs without sacrificing performance. The dependency on large, high-quality datasets to effectively train the model may pose challenges in cases where data availability is restricted or unreliable. Future work could explore methods to mitigate these issues, such as developing more efficient encoding schemes or employing data augmentation techniques to enhance model performance with smaller or noisier datasets.

## Data Availability

The original contributions presented in the study are included in the article/supplementary material, further inquiries can be directed to the corresponding author.
